# Supplementary material for: Placental network differences among obstetric syndromes identified with an integrated multiomics approach
Source: Commun Biol. 2025 Aug 18;8:1239. doi: 10.1038/s42003-025-08631-6 (PMC12361440; doi:10.1038/s42003-025-08631-6)
Supplement: Supplementary file 1 — Supplementary Materials [file 42003_2025_8631_MOESM1_ESM.pdf]

Table of Contents

Supplementary Figure 1. Gene expression across placental cell types 1

Supplementary Figure 2. No difference in cell type composition in FGR placentas...2

Supplementary Figure 3. No difference in cell type composition in PTD placentas...3

Supplementary Figure 4. Cell type composition differences in FGR+HDP placentas. 4

Supplementary Figure 5. Cell type composition differences in PE placentas. 5

Supplementary Figure 6. CLR-transformed cell type distributions in FGR+HDP placentas 6

Supplementary Figure 7. CLR-transformed cell type distributions in PE placentas. 7

Supplementary Figure 8. Correlation matrices of common confounders. 8

Supplementary Figure 9. Effect sizes are small for cofactors 9

Supplementary Figure 10. Gestational weeks at delivery effect size is comparable between fetal sexes. 10

Supplementary Figure 11. Random simulation of overlap of analytes significantly regulated by gestational age at delivery between two groups 11

Supplementary Figure 12. Effect size of significant interomics correlations is large across all obstetric conditions. 12

Supplementary Figure 13. FGR+HDP had the densest and least structured network...13

Supplementary Figure 14. Control interomics communities 15

Supplementary Figure 15. FGR interomics communities. 15

Supplementary Figure 16. PTD interomics communities 17

Supplementary Figure 17. FGR+HDP interomics community 18

Supplementary Figure 18. PE interomics communities. 18

Supplementary Figure 19. Cannot distinguish between FGR+HDP and other obstetric conditions using all measured analytes 19

Supplementary Table 2. Demographic, social history, pregnancy, and delivery characteristics composition between female and male fetuses 23

Supplementary Table 3. Number of analytes observed versus passing cutoffs by data type 23

Supplementary Table 4. FGR cell type composition. 24

Supplementary Table 5. PTD cell type composition. **25**

Supplementary Table 6. FGR+HDP cell type composition. **26**

Supplementary Table 7. PE cell type composition. **27**

Supplementary Table 8. Variance inflation factors for GLM features in female and male fetuses **28**

Supplementary Table 9. Network and partition quality metrics and overview after downsampling **28**

Supplementary Table 10. Key resources: software and algorithms. **29**

Supplementary References **30**

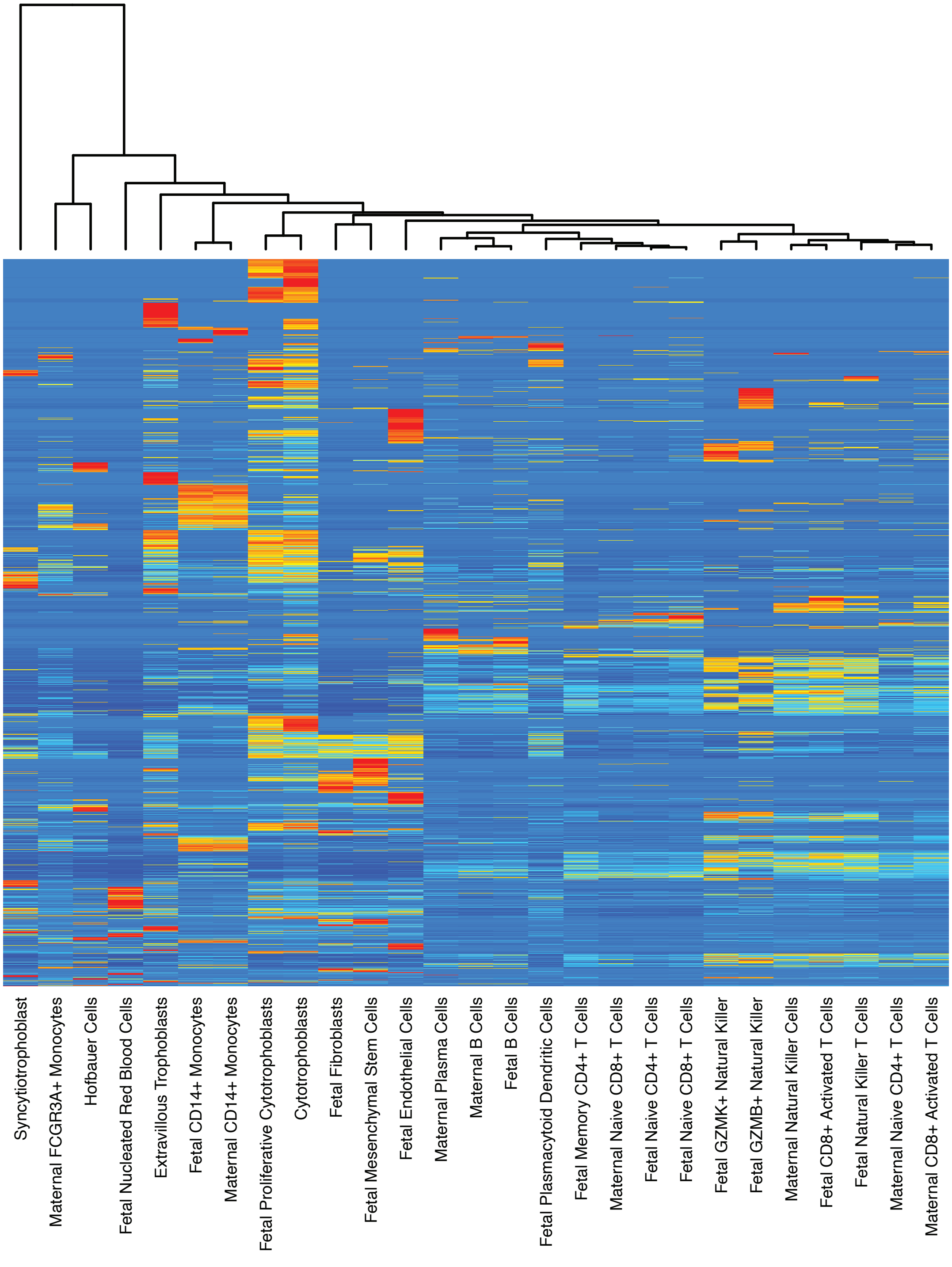

Supplementary Figure 1. Gene expression across placental cell types. Cell type cluster map of cell types (x-axis) and gene expression (y-axis). The color indicates the level of gene expression within a given cell type with red indicating high level of expression and blue indicating low level of expression. Hierarchical clustering was performed to group both the cell types for similarity of gene expression. This was generated from the cell-type signature matrix was generated using a single-cell RNA sequencing reference sample[1] normalized to counts per million (CPM), batch corrected using “S-mode” and permuted 100 times to assess statistical significance. This was generated by CIBERSORTx.

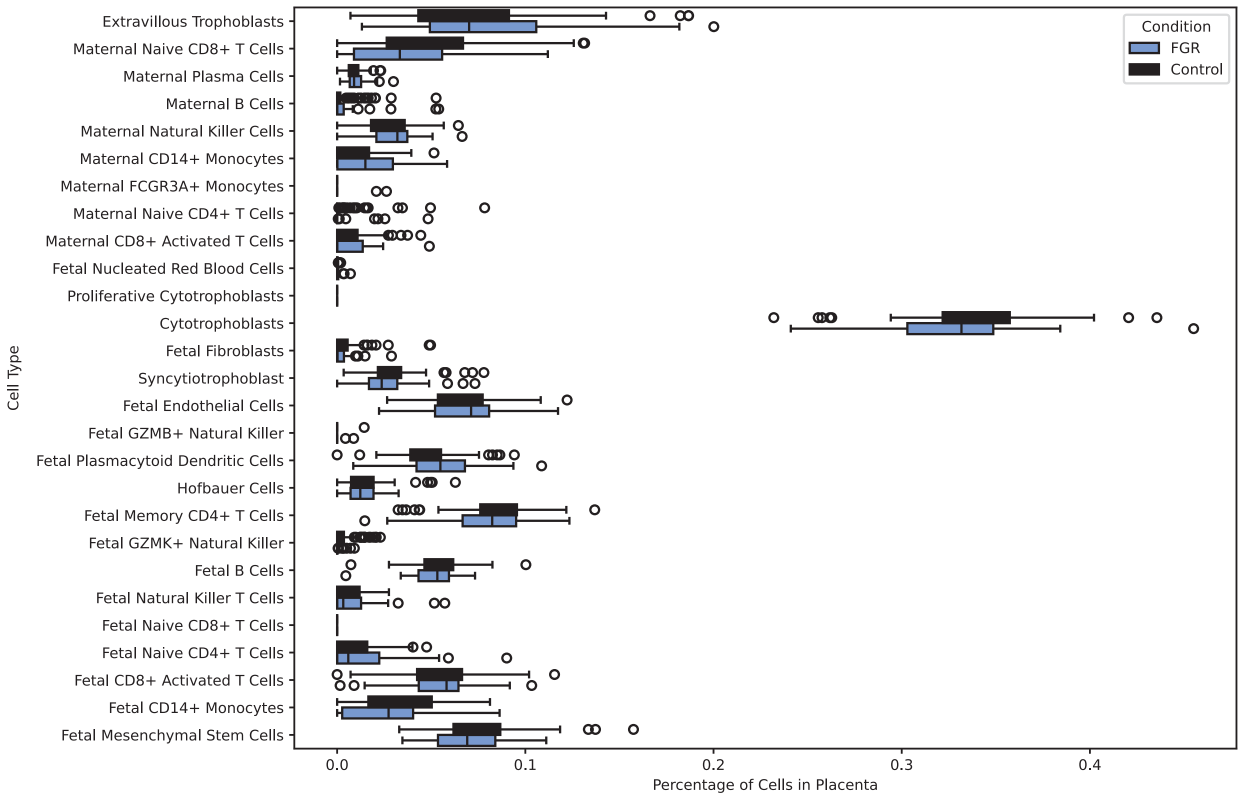

Supplementary Figure 2. No difference in cell type composition in FGR placentas. Boxplots of cell type percentages in FGR (blue) compared to control (black) placentas. Each box displays the interquartile range (IQR) from 25th percentile (Q1) to 75th percentile (Q3). The line in each box is the median. Whiskers extend to 1.5 times the IQR and the circles outside the whiskers are outliers. The cell type percentages displayed were not significantly different ($p>0.05$). The p-values were calculated by Kolmogorov-Smirnov tests followed by Benjamini-Hochberg multiple hypothesis corrections.

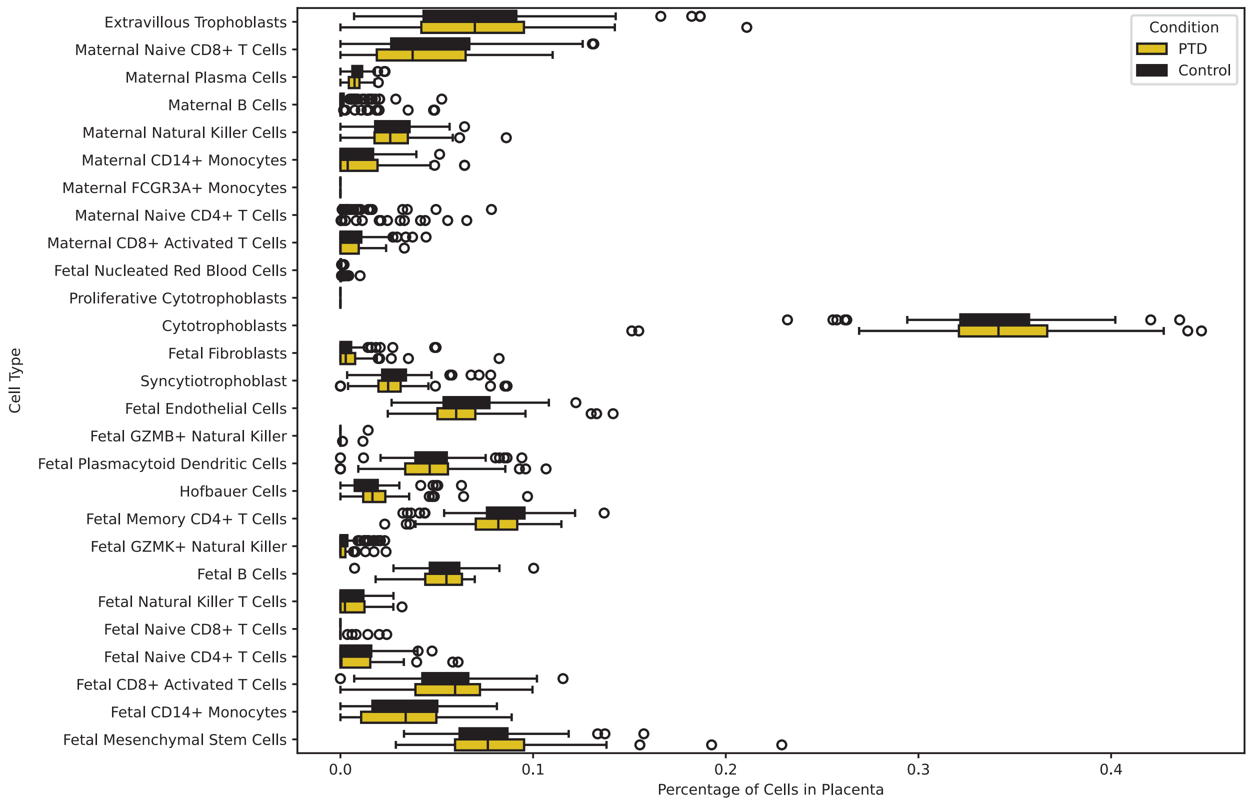

Supplementary Figure 3. No difference in cell type composition in PTD placentas. Boxplots of cell type percentages in PTD (yellow) compared to control (black) placentas. Each box displays the IQR from 25th percentile (Q1) to 75th percentile (Q3). The line in each box is the median. Whiskers extend to 1.5 times the IQR and the circles outside the whiskers are outliers. The cell type percentages displayed were not significantly different (p>0.05). The p-values were calculated by Kolmogorov-Smirnov tests followed by Benjamini-Hochberg multiple hypothesis corrections.

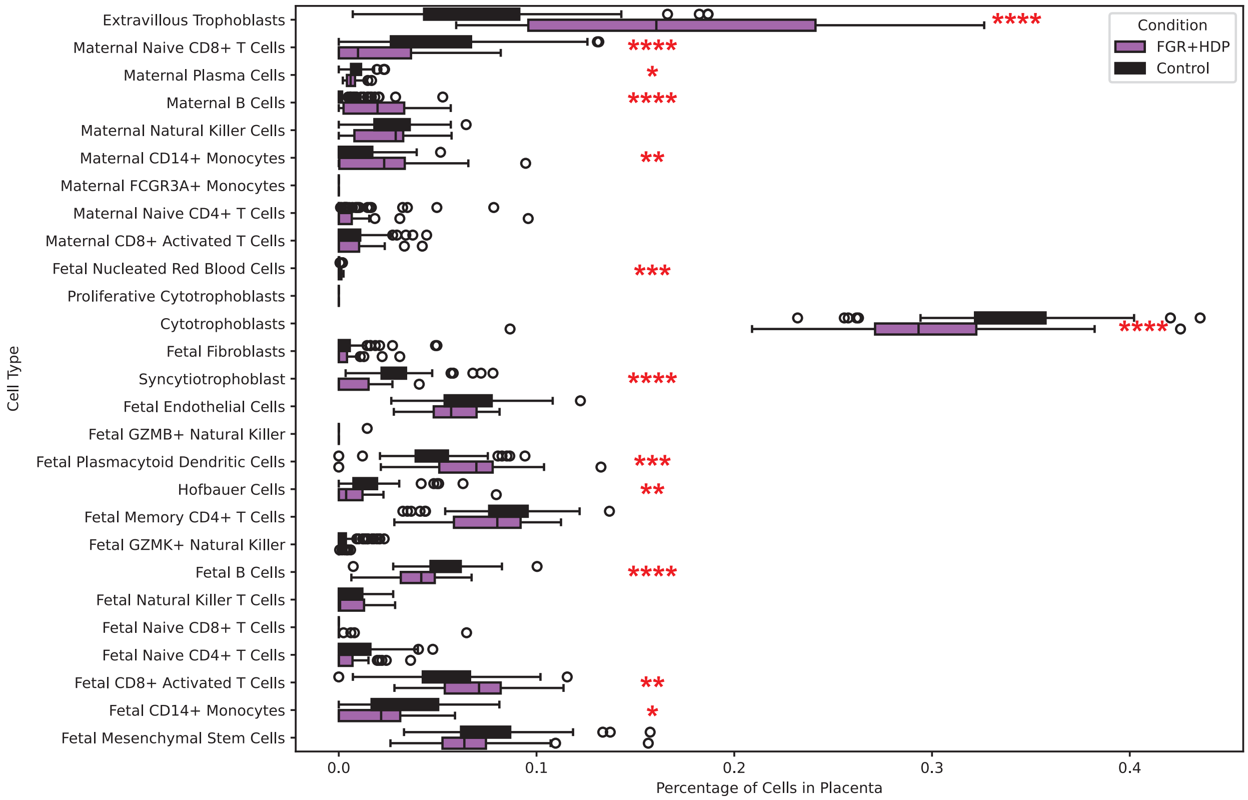

Supplementary Figure 4. Cell type composition differences in FGR+HDP placentas. Boxplots of cell type percentages in FGR+HDP (purple) compared to control (black) placentas. Each box displays the IQR from 25th percentile (Q1) to 75th percentile (Q3). The line in the box is the median. Whiskers extend to 1.5 times the IQR and the circles outside the whiskers are outliers. A subset of the cell type are significantly different (p>0.05). The p-values were calculated by Kolmogorov-Smirnov tests followed by Benjamini-Hochberg multiple hypothesis corrections. *p<0.05, **p<0.01, ***p<0.001, ****p<0.0001.

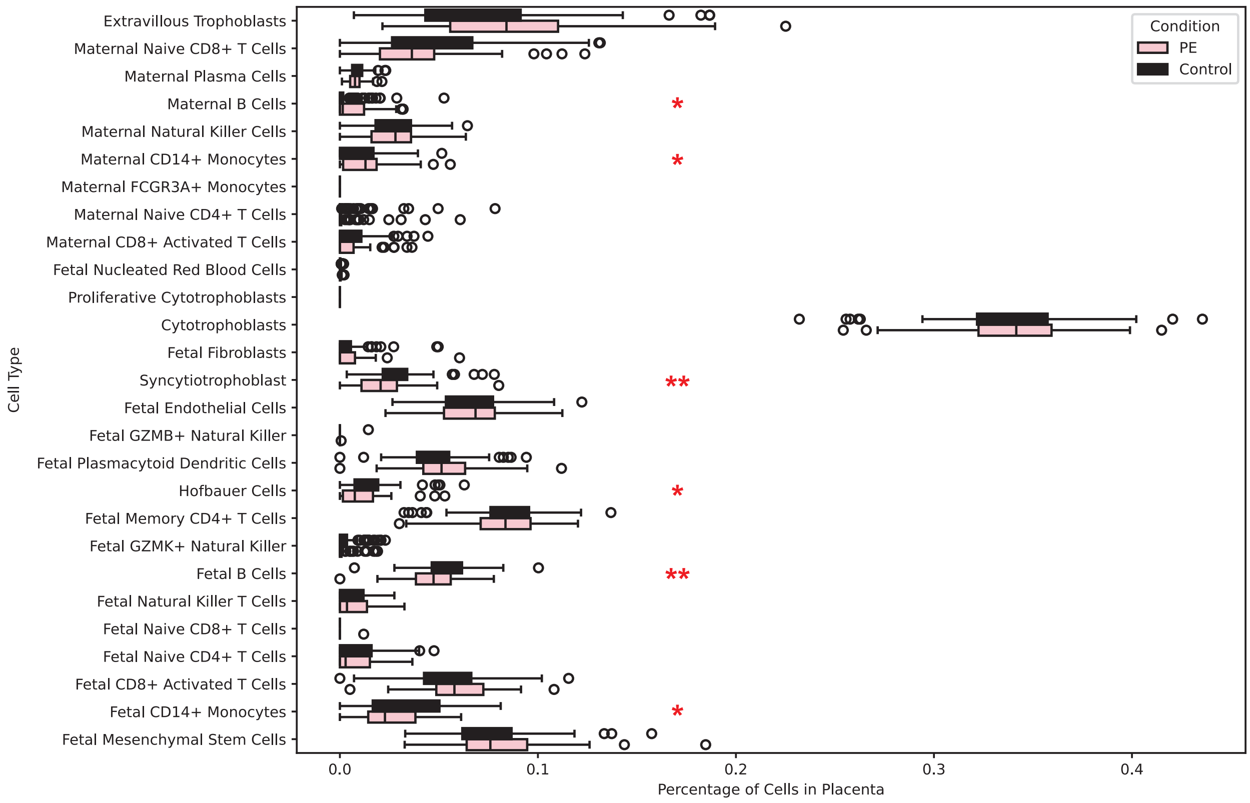

Supplementary Figure 5. Cell type composition differences in PE placentas. Boxplots of cell type percentages in FGR+HDP (pink) compared to control (black) placentas. Each box displays the IQR from 25th percentile (Q1) to 75th percentile (Q3). The line in the box is the median. Whiskers extend to 1.5 times the IQR and the circles outside the whiskers are outliers. A subset of the cell type are significantly different (p>0.05). The p-values were calculated by Kolmogorov-Smirnov tests followed by Benjamini-Hochberg multiple hypothesis corrections. *p<0.05, **p<0.01.

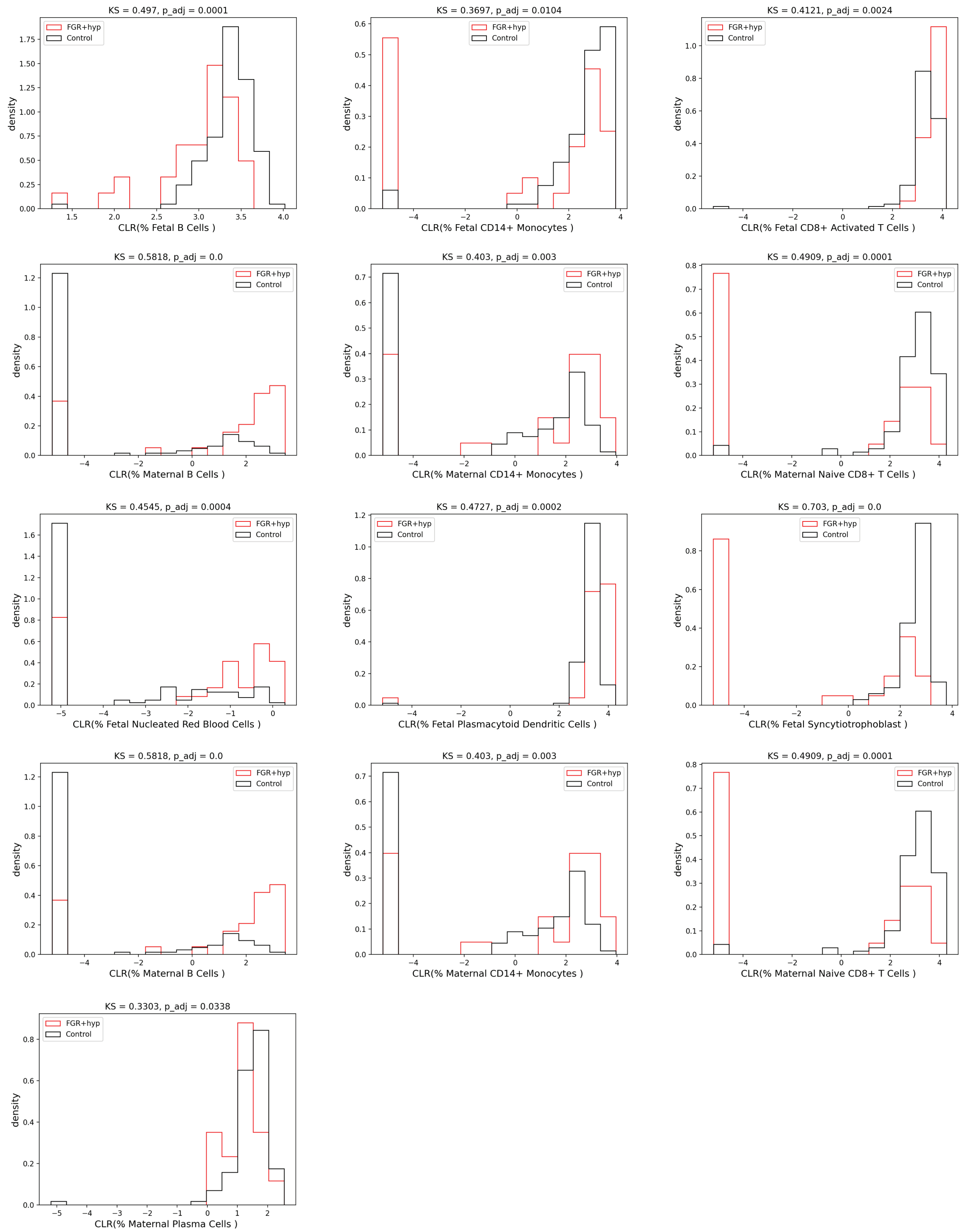

Supplementary Figure 6. CLR-transformed cell type distributions in FGR+HDP placentas. Histograms of clr-transformed cell type composition in FGR+HDP (red) and control (black) placentas. The p-values were calculated by Kolmogorov-Smirnov tests followed by Benjamini-Hochberg multiple hypothesis corrections.

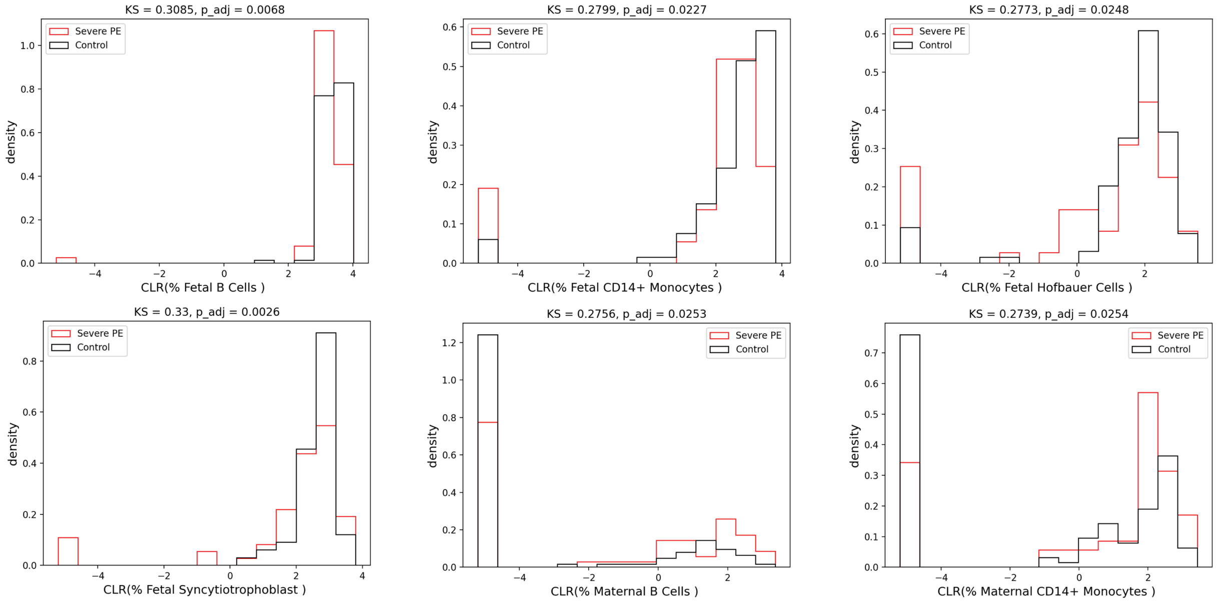

Supplementary Figure 7. CLR-transformed cell type distributions in PE placentas. Histograms of clr-transformed cell type composition in PE (red) and control (black) placentas. The p-values were calculated by Kolmogorov-Smirnov tests followed by Benjamini-Hochberg multiple hypothesis corrections.

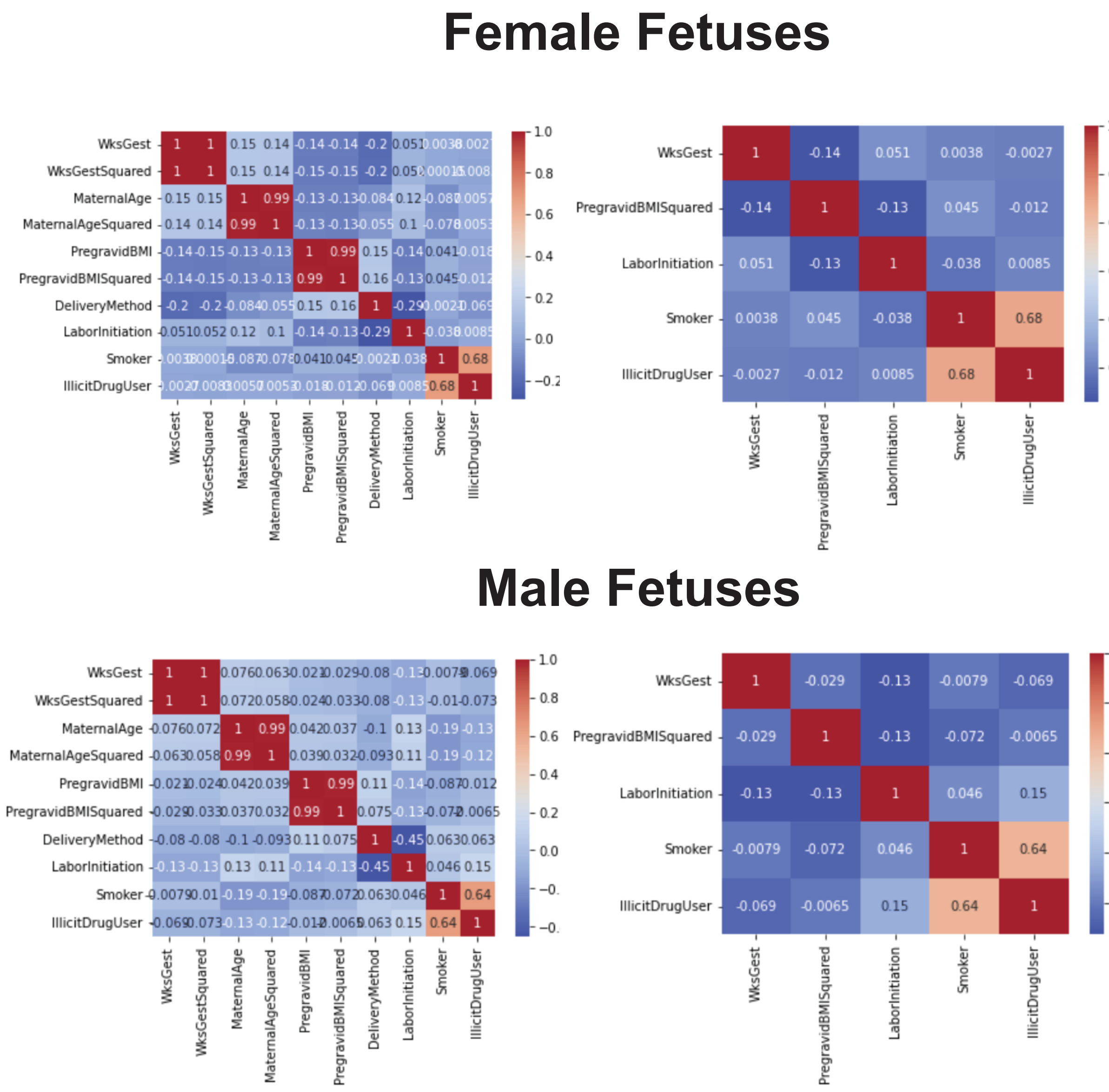

Supplementary Figure 8. Correlation matrices of common confounders. Correlation matrices to evaluate common confounders in female (top) and male (bottom) fetuses. Two matrices are displayed for each sex: one with all the confounding variables considered (left) and one containing only the variables used in the generalized linear models (GLM; right). The color indicates the strength of the correlation with dark blue indicating no correlation (0) and dark red indicating perfect correlation (1).

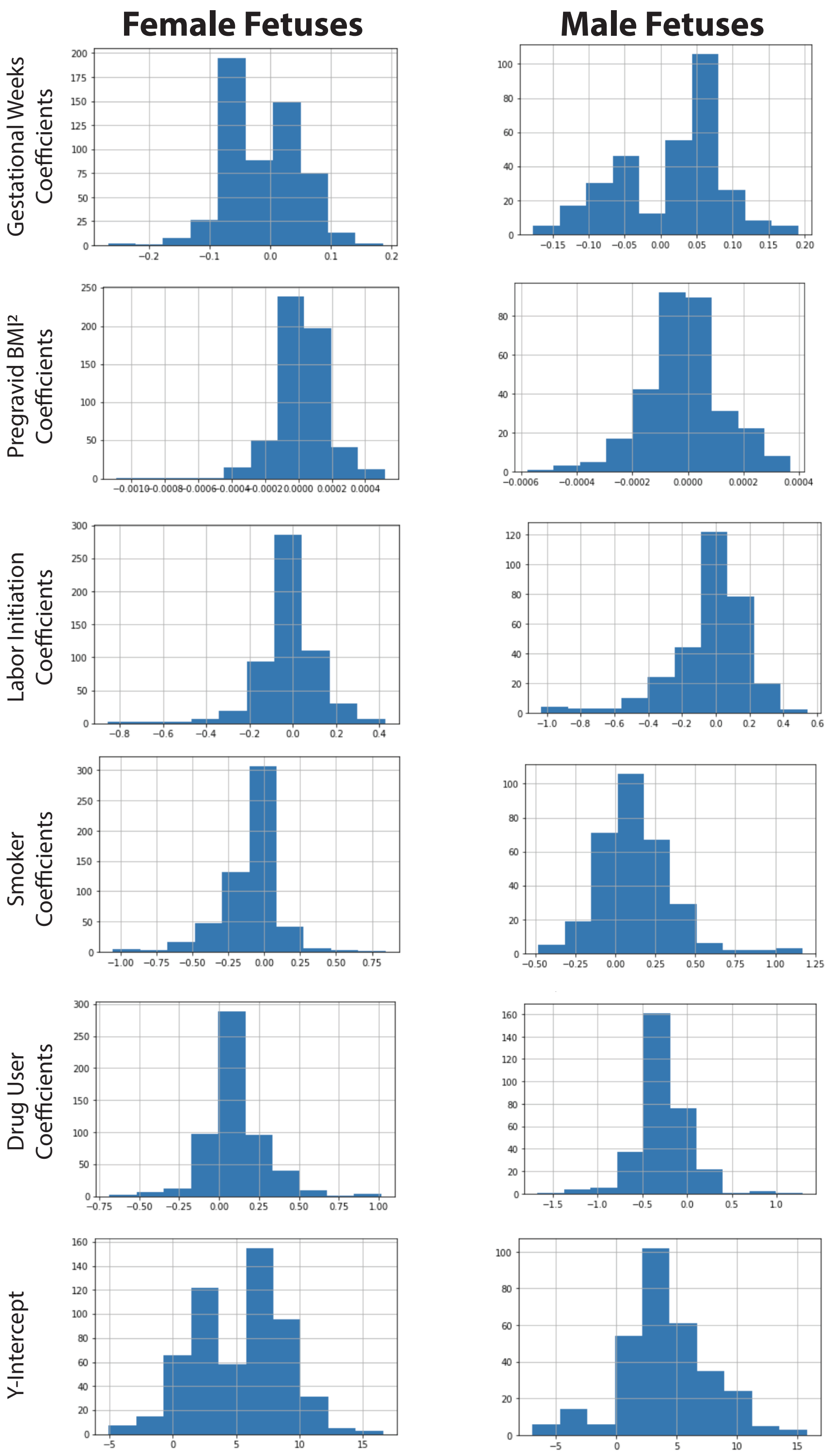

Supplementary Figure 9. Effect sizes are small for cofactors. Histograms of the β-coefficients for each variable in the GLMs for female (left) and male (right) fetuses. The β-coefficients were limited to those in GLMs whose gestational weeks at delivery was significantly regulated (FDR<0.05) following Benjamini-Hochberg multiple hypothesis adjustment

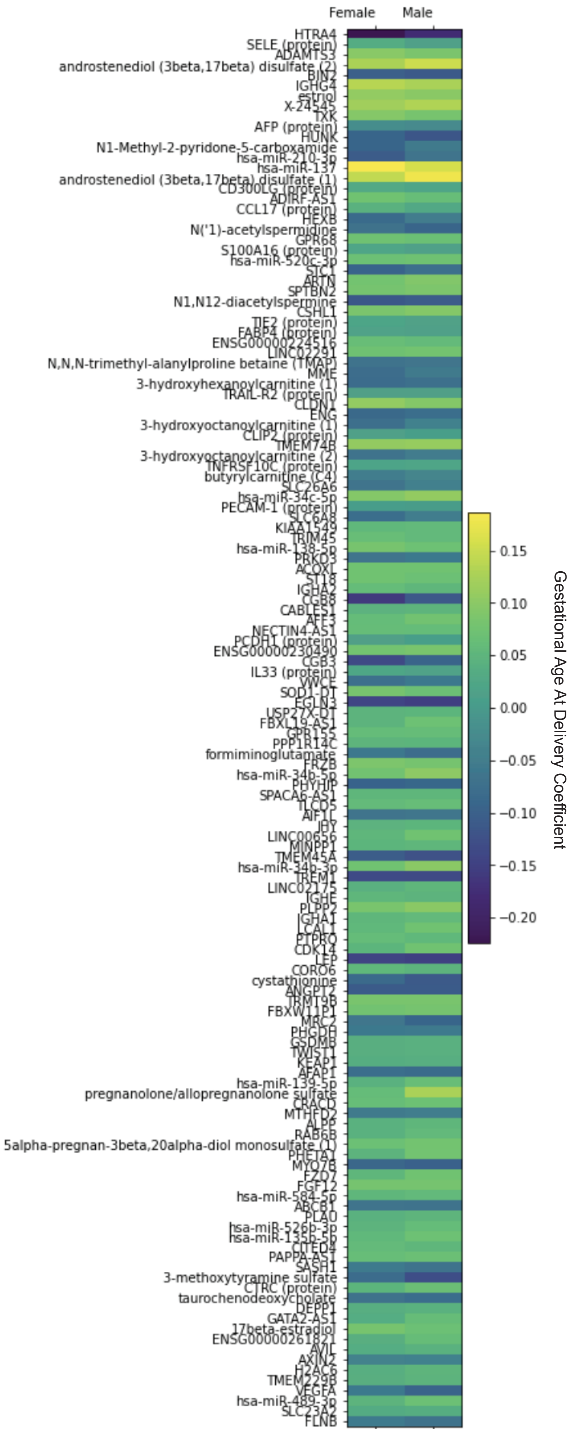

Supplementary Figure 10. Gestational weeks at delivery effect size is comparable between fetal sexes. Heatmap of the β-coefficient of the gestational weeks at delivery for the GLMs of analytes that are significantly regulated (FDR<0.05) by this variable in both fetal sexes following Benjamini-Hochberg multiple hypothesis adjustment. Purple indicates a low, negative β-coefficient and yellow indicates a high, positive β-coefficient.

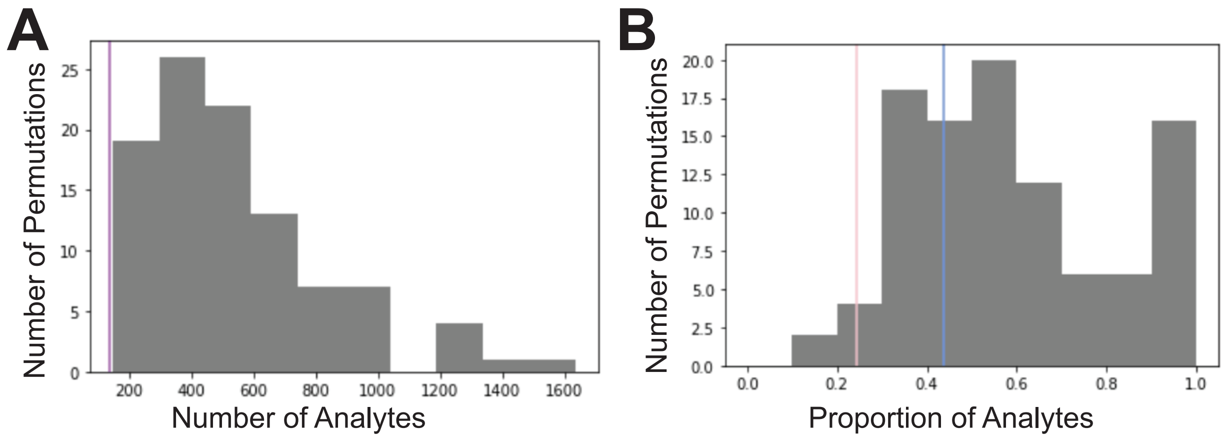

Supplementary Figure 11. Random simulation of overlap of analytes significantly regulated by gestational age at delivery between two groups. A hundred iterations were performed in which each sample was assigned to one of two groups, GLMs were fit for each analyte in each group, and the overlap in the analytes significantly regulated (FDR<0.05) by gestational weeks at delivery in both random groups following Benjamini-Hochberg multiple hypothesis correction was assessed. (**A**) Histogram (bin size=100) of the number of significant analytes in common between the two groups across all permutations. The purple vertical line (y=136) is what is observed when comparing female and male fetuses. As this falls below the range of anything observed by random chance this means FDR<0.01 for this observation. (**B**) Histogram (bin size=0.1) of the proportion of analytes significantly regulated by gestational weeks at delivery in a random group that was observed to be in common with the second random group across all permutations. The vertical pink (y=0.244) and blue (y=0.439) lines represent the proportion of significant analytes observed in female and male fetuses respectively. Where these fall within the range of random permutations indicates a FDR<0.02 and FDR=0.14 for the proportion of significant analytes in female fetuses that also are significantly regulated in male fetuses and vice-versa.

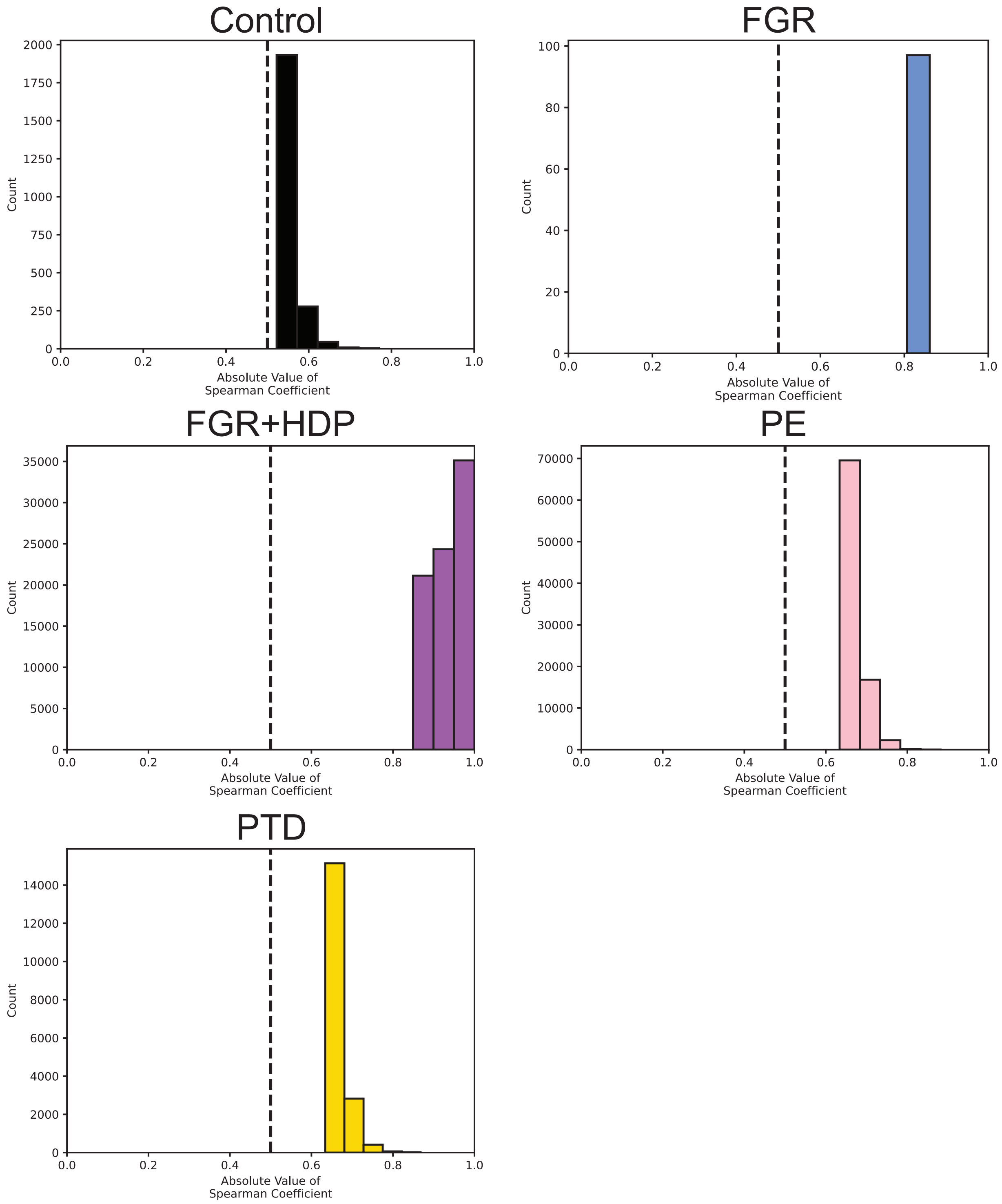

Supplementary Figure 12. Effect size of significant interomics correlations is large across all obstetric conditions. Histograms of the absolute value of the spearman coefficients of significant ($p<0.05$) interomics correlations following Bonferroni correction. The vertical dotted line ($y=0.5$) is the boundary for large effect size indicating that anything to the right of the line has a large effect size. This was reported for control (black), FGR (blue), FGR+HDP (purple), PE (pink), and PTD (gold) interomics networks.

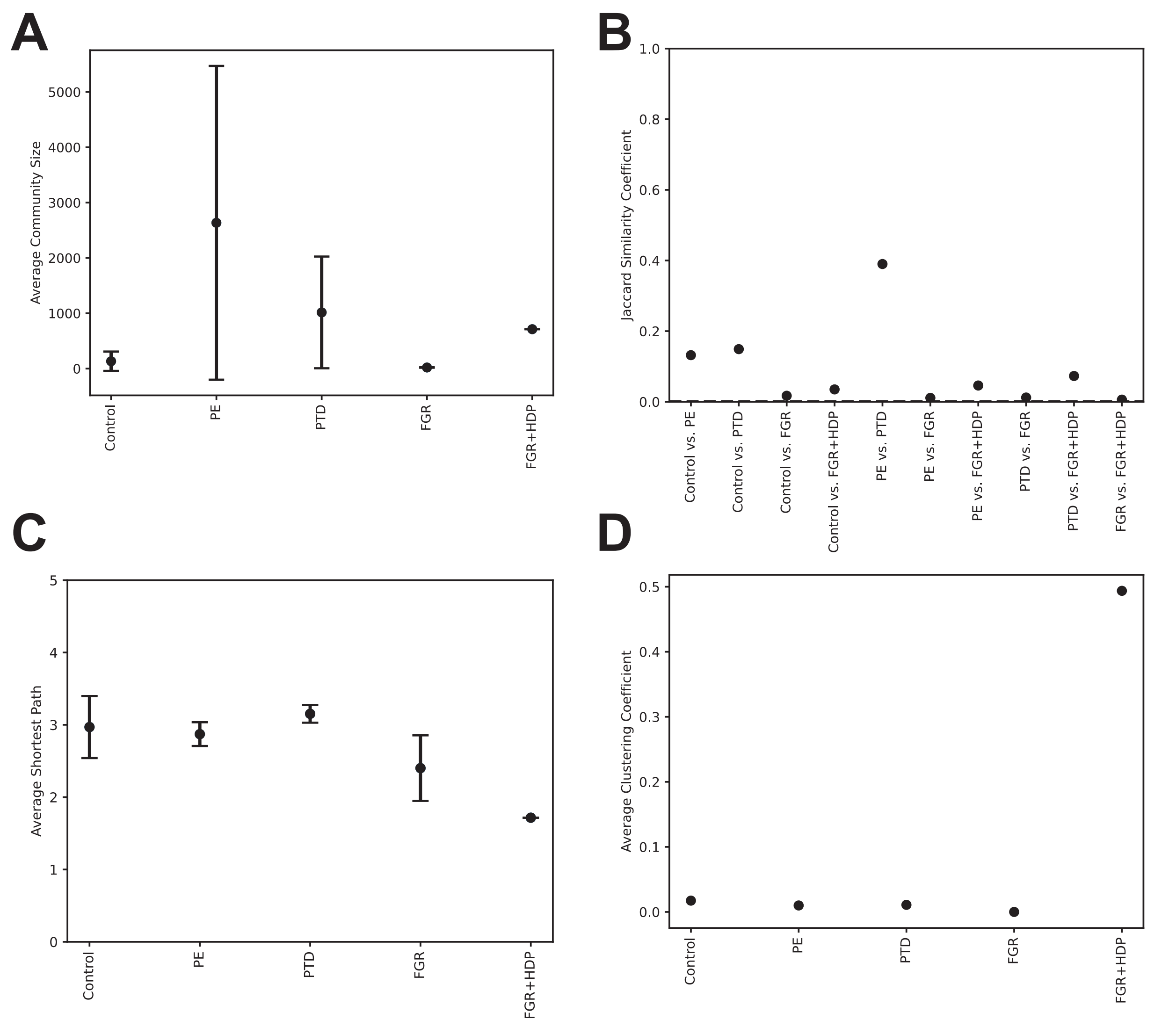

Supplementary Figure 13. FGR+HDP had the densest and least structured network. Evaluations of the interomics network structure and composition across obstetric conditions. (**A**) Dot plot with error bars of mean and standard deviation of the average community size. (**B**) Dot plots of the Jaccard Similarity Coefficient between all possible pairwise combinations of conditions. (**C**) Dot plot with error bars of the mean and standard deviation of the average shortest path within a community. (**D**) Dot plot of the average clustering coefficient.

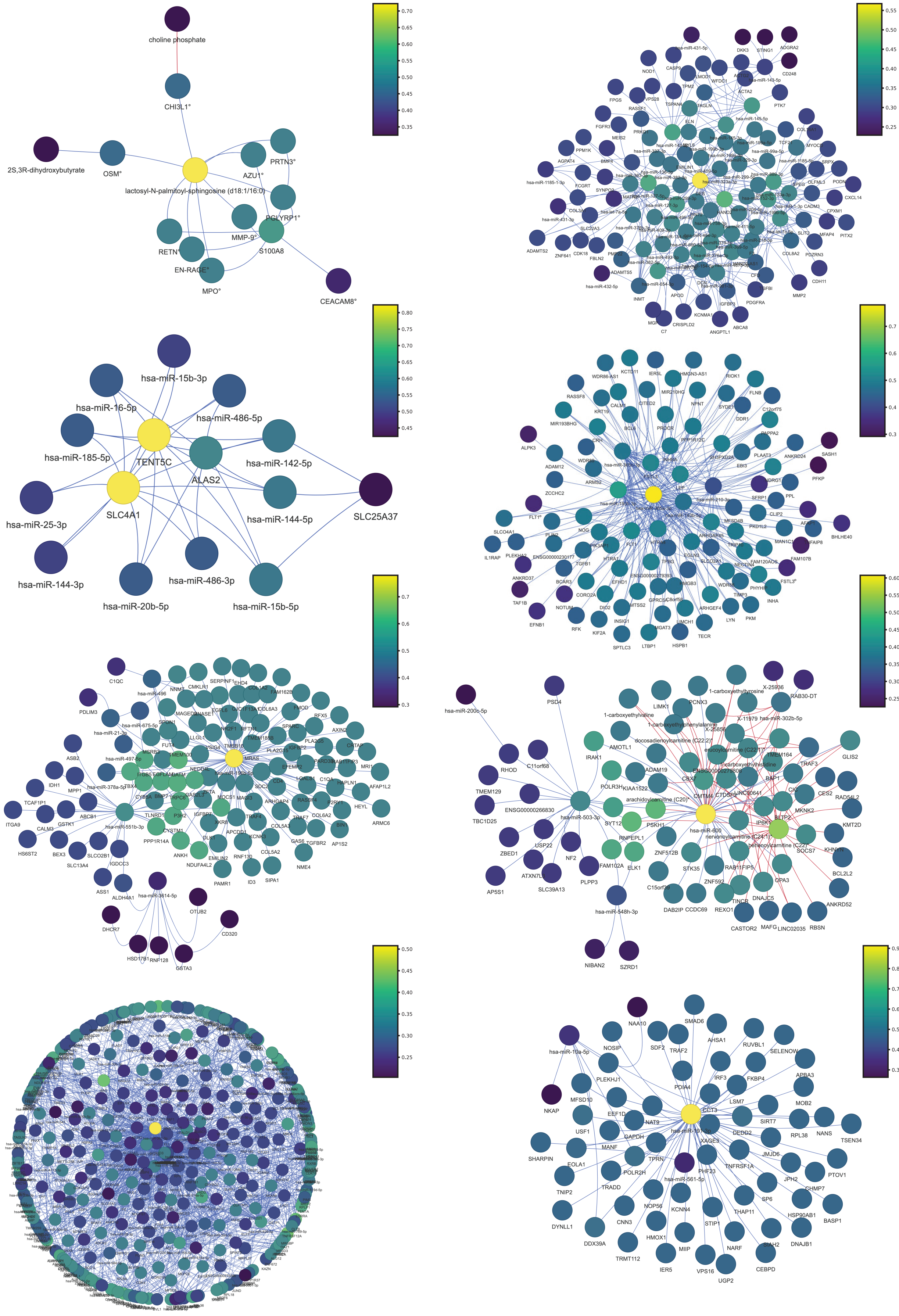

choline phosphate
CHI3L1°
2S,3R-dihydroxybutyrate
OSM°
lactosyl-N-palmitoyl-sphingosine (d18:1/16:0)
AZU1°
PRTN3°
PGLYRP1°
MMP-9°
S100A8
RETN°
EN-RAGE°
MPO°
CEACAM8°
hsa-miR-15b-3p
hsa-miR-16-5p
hsa-miR-486-5p
hsa-miR-185-5p
TENT5C
hsa-miR-142-5p
ALAS2
SLC4A1
hsa-miR-25-3p
hsa-miR-144-5p
SLC25A37
hsa-miR-144-3p
hsa-miR-486-3p
hsa-miR-20b-5p
hsa-miR-15b-5p

Supplementary Figure 14. Control interomics communities. All control interomics communities that have ten or more nodes. Analyte names are listed below each node. A ° was added to the end of protein names (e.g. FLT1°) to distinguish proteins from mRNAs. The edge color indicates the directionality of the correlation with blue representing a positive correlation and red representing a negative correlation The node color indicates the closeness centrality with yellow indicating the most connected node and purple indicating the least connected node. A color bar is to the right of each community and was normalized to a scale of 0 to 1 for each community. The individual html files for each of these communities is available on the paper's GitHub repository.

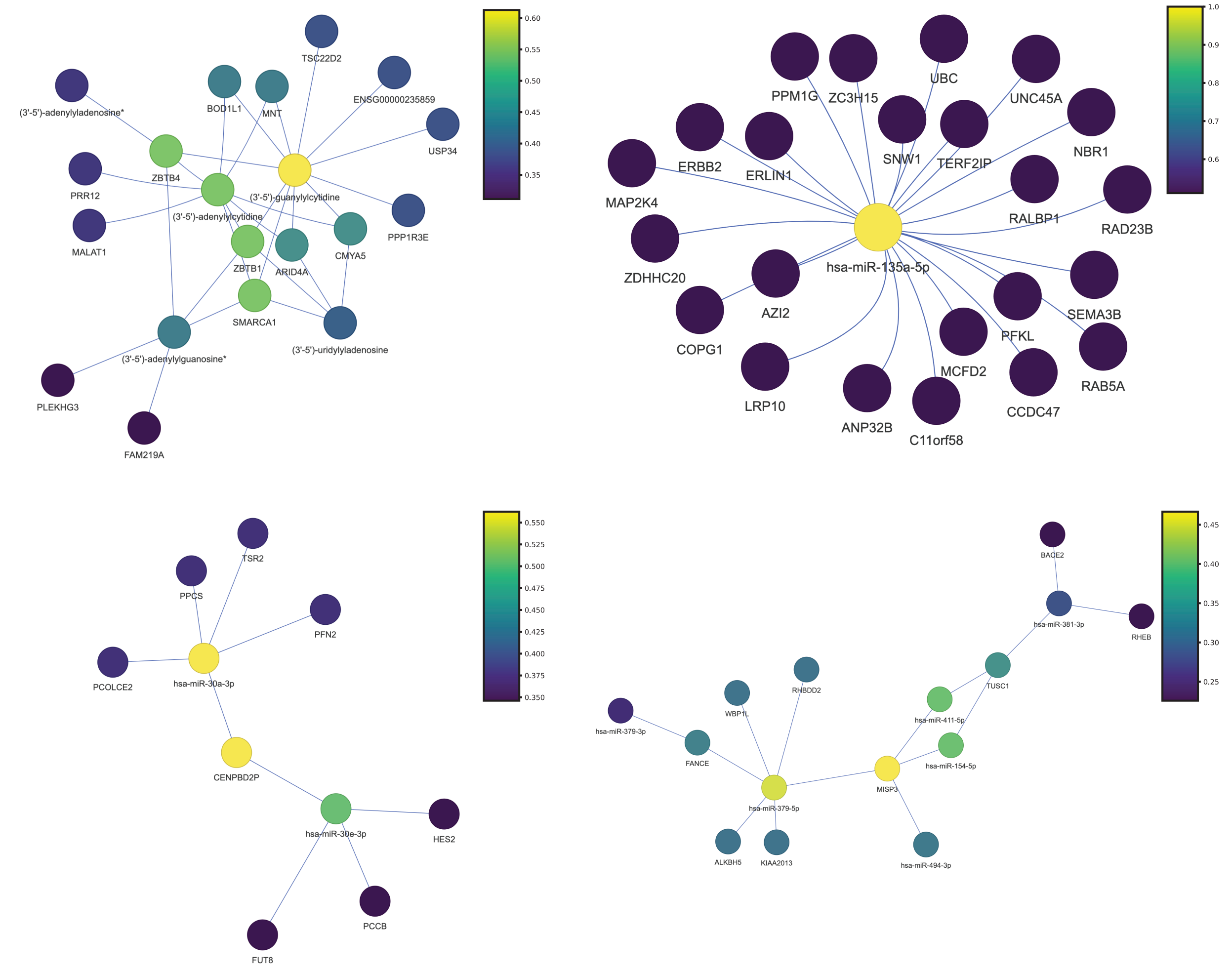

Supplementary Figure 15. FGR interomics communities. All FGR interomics communities that have ten or more nodes. Analyte names are listed below each node. A ° was added to the end of protein names (e.g. FLT1°) to distinguish proteins from mRNAs. The edge color indicates the directionality of the correlation with blue representing a positive correlation and red representing a negative correlation The node color indicates the closeness centrality with yellow indicating the most connected node and purple indicating the least connected node. A color bar is to the right of each community and was normalized to a scale of 0 to 1 for each community. The individual html files for each of these communities is available on the paper's GitHub repository.

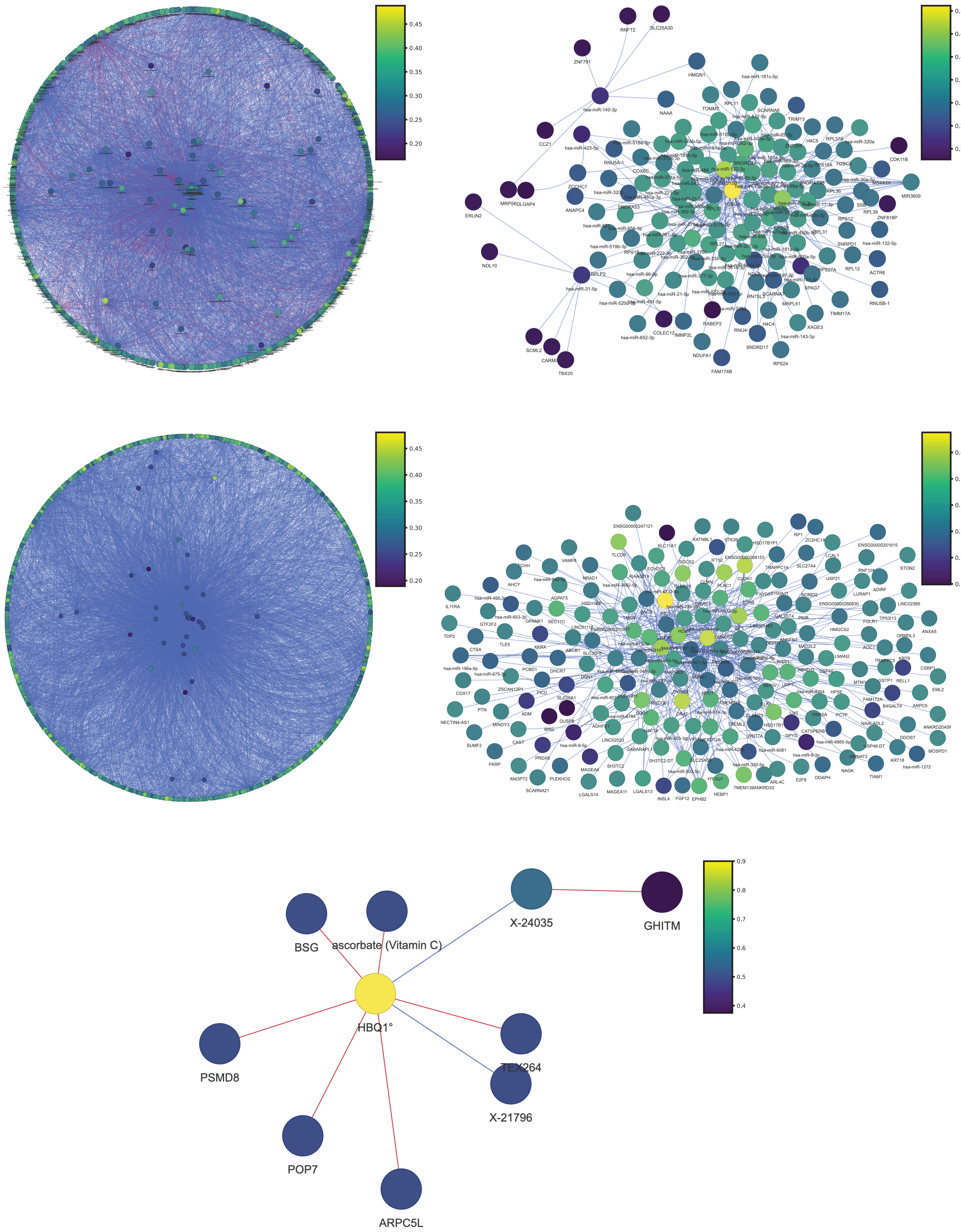
BSG
ascorbate (Vitamin C)
X-24035
GHITM
HBQ1°
PSMD8
TEX264
X-21796
POP7
ARPC5L

Supplementary Figure 16. PTD interomics communities. All PTD interomics communities that have ten or more nodes. Analyte names are listed below each node. A ° was added to the end of protein names (e.g. FLT1°) to distinguish proteins from mRNAs. The edge color indicates the directionality of the correlation with blue representing a positive correlation and red representing a negative correlation The node color indicates the closeness centrality with yellow indicating the most connected node and purple indicating the least connected node. A color bar is to the right of each community and was normalized to a scale of 0 to 1 for each community. The individual html files for each of these communities is available on the paper's GitHub repository.

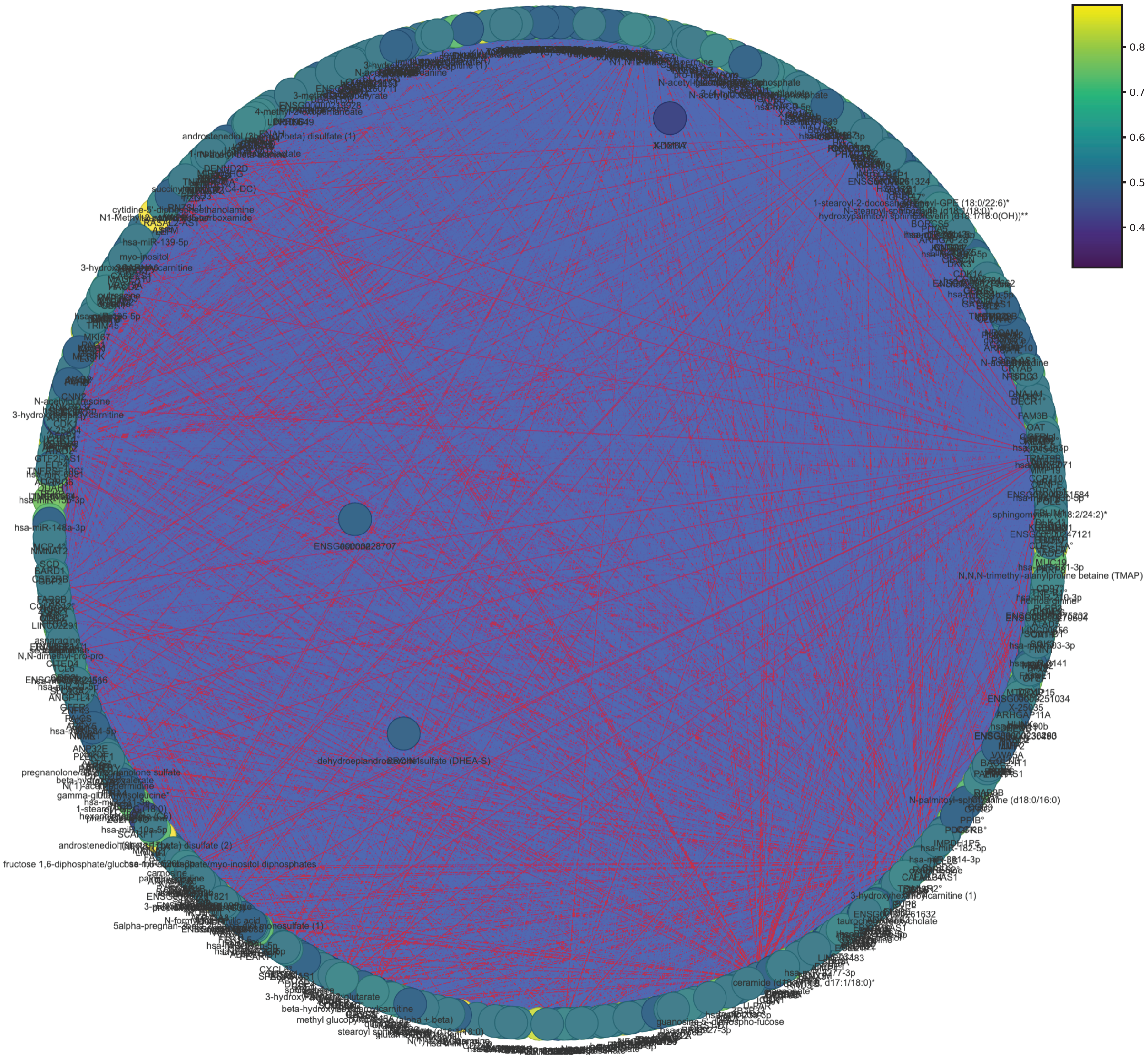

Supplementary Figure 17. FGR+HDP interomics community. The only FGR+HDP interomics community that has ten or more nodes. Analyte names are listed below each node. A ° was added to the end of protein names (e.g. FLT1°) to distinguish proteins from mRNAs. The edge color indicates the directionality of the correlation with blue representing a positive correlation and red representing a negative correlation The node color indicates the closeness centrality with yellow indicating the most connected node and purple indicating the least connected node. The color bar was normalized to a scale of 0 to 1 for each community. The individual html files for this community is available on the paper's GitHub repository.

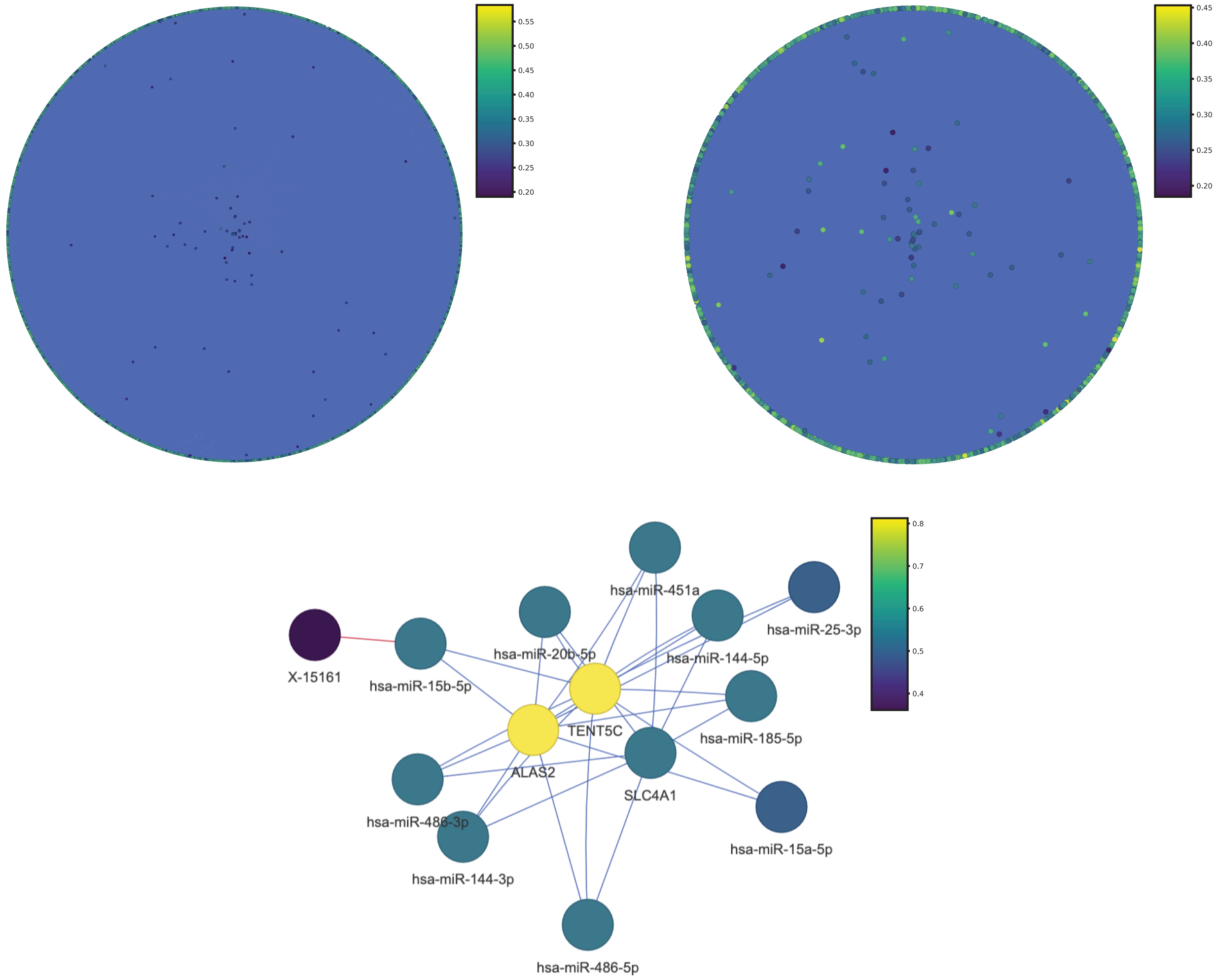

Supplementary Figure 18. PE interomics communities. All PE interomics communities that have ten or more nodes. Analyte names are listed below each node. A ° was added to the end of protein names (e.g. FLT1°) to distinguish proteins from mRNAs. The edge color indicates the directionality of the correlation with blue representing a positive correlation and red representing a negative correlation The node color indicates the closeness centrality with yellow indicating the most connected node and purple indicating the least connected node. A color bar is to the right of each community and was normalized to a scale of 0 to 1 for each community. The individual html files for each of these communities is available on the paper's GitHub repository.

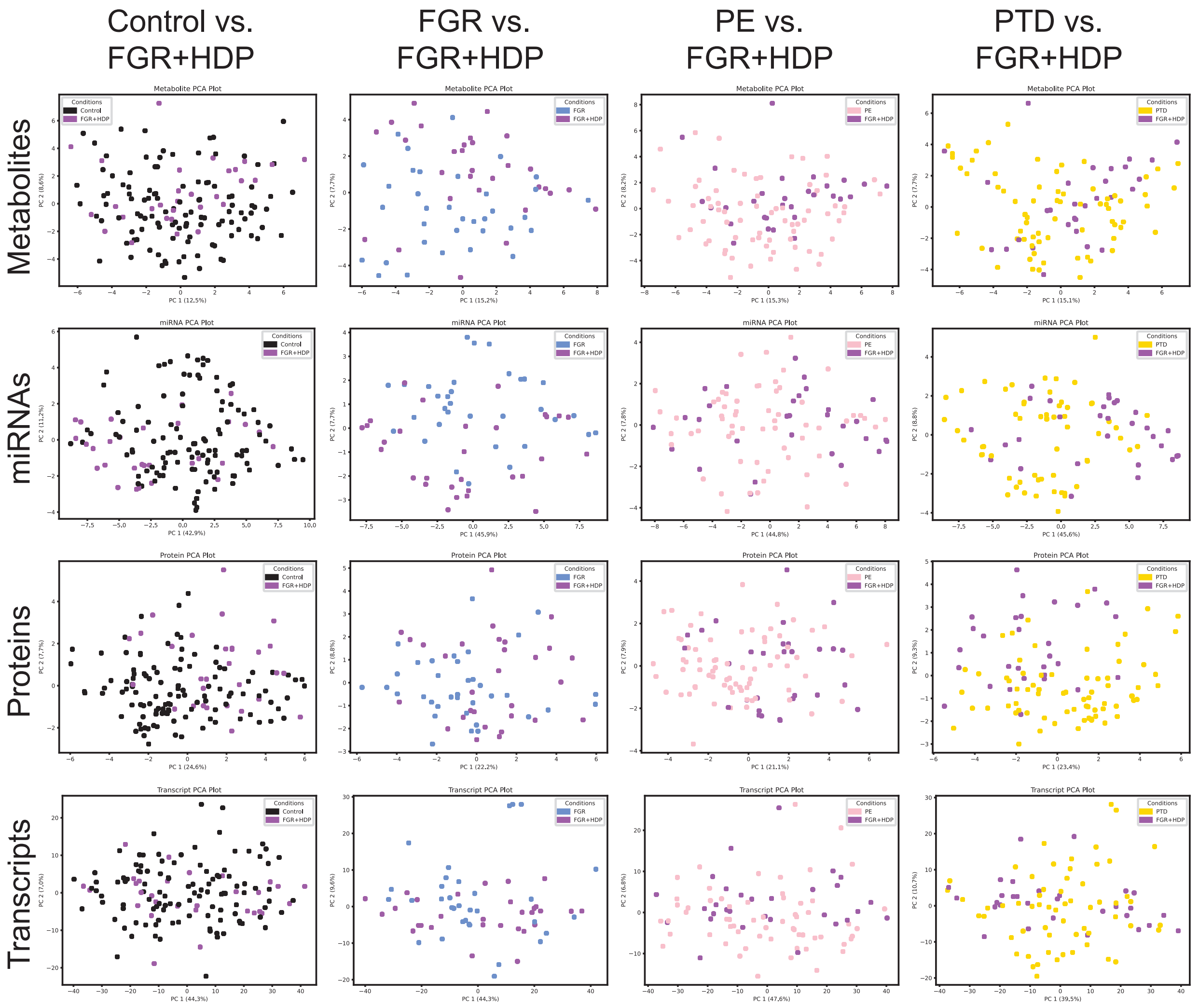

Supplementary Figure 19. Cannot distinguish between FGR+HDP and other obstetric conditions using all measured analytes. Principal Component Analyses (PCA) first two dimensions were plotted using all metabolites (first row), miRNAs (second row), proteins (third row), and mRNA transcripts (fourth row). This was done for FGR+HDP (purple) alongside each of the other obstetric conditions: Control (black; column one), FGR (blue; column two), PE (pink, column three), and PTD (gold, column four). Each dot represents a placenta and the color of the dot indicates the condition to which it belongs.

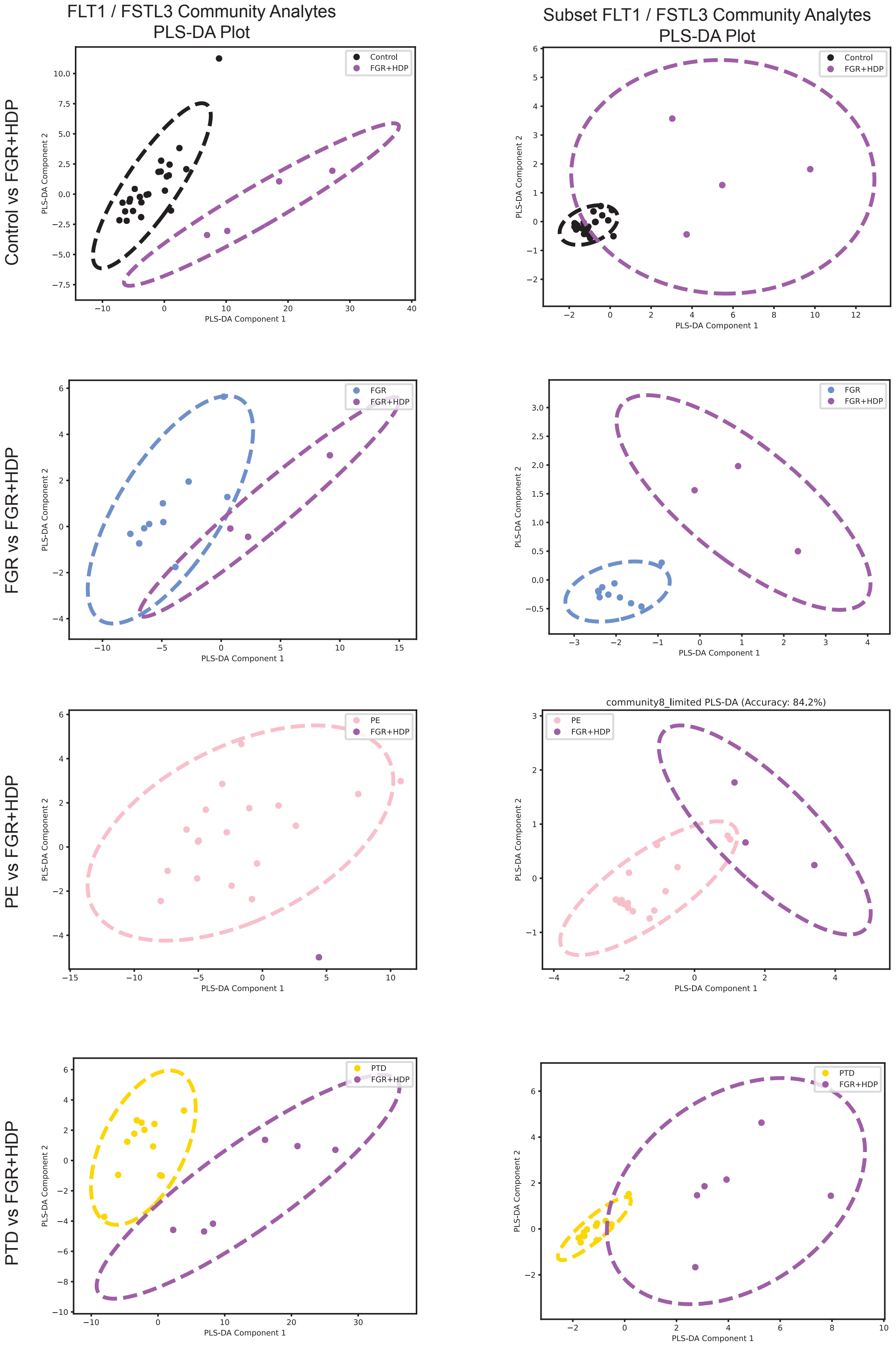

FLT1 / FSTL3 Community Analytes
PLS-DA Plot
Subset FLT1 / FSTL3 Community Analytes
PLS-DA Plot
Control vs FGR+HDP
FGR vs FGR+HDP
PE vs FGR+HDP
PTD vs FGR+HDP
community8_limited PLS-DA (Accuracy: 84.2%)
Control
FGR+HDP
FGR
PE
PTD
PLS-DA Component 1
PLS-DA Component 2

Supplementary Figure 20. FGR+HDP signature distinguishes it from placentas with overlapping clinical features. Partial Least Square-Discriminant Analysis (PLS-DA) of the FGR+HDP (purple) placentas plotted alongside the control (black; top panels), FGR (blue; middle top panels), PE (pink; middle bottom panels), and PTD (gold; bottom panels) for both all 100 analytes in *FLT1* / *FSTL3* community (left) and the twelve analytes – all mRNA transcripts - in the final FGR+HDP biosignature (right). Each dot represents a placenta with the color of the dot indicating the placenta's condition. The dotted line ellipses indicate the 95% confidence interval for each condition. The twelve analytes in the final FGR+HDP biosignature were selected for having the most distinctive differential expression across all pairwise comparisons of conditions as revealed by hierarchical clustering (Figure 4B).

Feature	Definition
**Demographic**	
Maternal Age (Years)	Maternal age at start of pregnancy (years); continuous variable
Pregravid BMI (kg/m2)	Pregravid Body Mass Index (BMI; kg/m2); continuous variable
Race	Reported race: Asian, Black, Native American, White, Multiracial, or not reported (missing); categorical variable
Ethnicity, Hispanic	Reported ethnicity: Non-Hispanic or Unknown (0) or Hispanic (1); binary variable
**Social History**	
Smoking	Smoking status: not reported as smoking during pregnancy (0), or reported smoking during pregnancy (1)
Illicit Drug Use	Illicit drug status: no reported illicit drug use (0), or reported illicit drug use defined as cocaine use, substance abuse, marijuana use, maternal narcotic addiction at delivery, or drug withdrawal syndrome in newborn (1); binary variable
**Pregnancy Characteristics**	
Condition	Obstetric condition associated with the pregnancy: Control Term (delivery $\geq$37 weeks gestation, $\geq$10th percentile birthweight by gestational age, no pregnancy complications), Fetal Growth Restriction (FGR; <3rd percentile birthweight by gestational age; no hypertensive disorders); Fetal Growth Restriction with Precnancy Related Hypertension (FGR+HDP; <3rd percentile birthweight by gestational age with hypertensive disorder); Severe Preeclampsia (PE; severe features defined according to the American College of Obstetricians and Gynecologists guidelines); Spontaneous Preterm Delivery (PTD; delivery <37 weeks gestation with spontaneous labor present); categorical variable
Parity	Number of times a person has given birth to a fetus older than 24 weeks of gestation prior to the current pregnancy: Nulliparity (0 births), Multiparous (1 - 4 births) or Grand Multiparous (5+ births); categorical variable
**Delivery Charecteristics**	
Delivery Method	Reports method of delivery as being vaginal (0) or caesarean section (C-Section; 1); binary variable
Labor Initiation	Reports if spontaneous laber was present (0) or absent (1) at delivery, those without spontaneous labor may have been induced or had no labor at delivery; binary variable
Birthweight, g	Birthweight recorded at delivery (g); continuous variable
Gestational Age at Delivery, Weeks	Gestational age at delivery (weeks); continuous variable

Supplementary Table 1. Feature definitions for demographic, social history, pregnancy, and delivery characteristics.

	p-value
	Female vs Male Fetus
**Demographics**	
Maternal Age, Years	p=0.15
Pregravid BMI, kg/m2	p<0.01
Race	p<0.05
Ethnicity	p=0.97
**Social History**	
Smoking	p=0.09
Illicit Drug Use	p=0.53
**Pregnancy Characteristics**	
Condition	p<0.01
Parity	NA
**Delivery Characteristics**	
Delivery Method	p=0.06
Labor Initiation	p<0.01
Birthweight, g	p=0.32
Gestational Weeks at Delivery	p=0.53

Supplementary Table 2. Demographic, social history, pregnancy, and delivery characteristics composition between female and male fetuses. Chi square test and students two tailed t-test were performed for categorical and continuous characteristics respectively.

Analyte	Number Observed	Number Passing Cutoffs
Metabolites	1032	865
miRNAs	2414	448
Proteins	452	343
Transcripts	51,174	9582
Total	52,658	11,238

Supplementary Table 3. Number of analytes observed versus passing cutoffs by data type.

Cell Type	KS Statistic	p-value	Group Mean (CLR)	Control Mean (CLR)	Group STD (CLR)	Control STD (CLR)	Group Mean (%)	Control Mean (%)	Group STD (%)	Control STD (%)	p-value Adjusted
Fetal Mesenchymal Stem Cells	0.184	0.287	3.616	3.693	0.300	0.286	0.070	0.076	0.020	0.022	0.535
Fetal CD14+ Monocytes	0.242	0.075	0.907	2.482	3.294	1.667	0.026	0.035	0.024	0.020	0.225
Fetal CD8+ Activated T Cells	0.096	0.945	3.247	3.262	0.743	0.914	0.054	0.054	0.021	0.020	1.000
Fetal Naive CD4+ T Cells	0.145	0.572	-0.486	-0.746	3.571	3.388	0.015	0.010	0.021	0.012	0.824
Fetal Naive CD8+ T Cells	0.061	1.000	-5.196	-5.196	0.000	0.000	0.000	0.000	0.000	0.000	1.000
Fetal Natural Killer T Cells	0.145	0.572	-1.271	-0.822	3.510	3.205	0.009	0.007	0.014	0.007	0.824
Fetal B Cells	0.149	0.540	3.286	3.364	0.450	0.307	0.051	0.054	0.013	0.014	0.821
Fetal GZMK+ Natural Killer	0.192	0.244	-3.769	-2.858	2.397	3.105	0.001	0.003	0.002	0.006	0.499
Fetal Memory CD4+ T Cells	0.213	0.154	3.691	3.828	0.438	0.248	0.078	0.085	0.025	0.018	0.377
Fetal Hofbauer Cells	0.092	0.959	1.615	1.500	1.425	1.816	0.014	0.015	0.009	0.011	1.000
Fetal Plasmacytoid Dendritic Cells	0.283	0.022	3.359	3.159	0.454	0.864	0.056	0.048	0.020	0.015	0.097
Fetal GZMB+ Natural Killer	0.061	1.000	-4.828	-5.130	1.496	0.690	0.000	0.000	0.002	0.001	1.000
Fetal Endothelial Cells	0.190	0.258	3.551	3.570	0.395	0.269	0.067	0.066	0.022	0.017	0.506
Fetal Syncytiotrophoblast	0.248	0.063	2.264	2.623	1.450	0.563	0.026	0.028	0.016	0.013	0.199
Fetal Fibroblasts	0.157	0.476	-2.136	-1.794	3.029	3.107	0.003	0.004	0.006	0.008	0.734
Fetal Cytotrophoblasts	0.229	0.105	5.195	5.229	0.120	0.097	0.328	0.339	0.040	0.032	0.280
Fetal Proliferative Cytotrophoblasts	0.061	1.000	-5.196	-5.196	0.000	0.000	0.000	0.000	0.000	0.000	1.000
Fetal Nucleated Red Blood Cells	0.240	0.078	-2.929	-3.827	2.204	1.868	0.001	0.000	0.001	0.000	0.226
Maternal CD8+ Activated T Cells	0.110	0.862	-2.254	-2.083	3.625	3.438	0.007	0.006	0.011	0.010	1.000
Maternal Naive CD4+ T Cells	0.078	0.992	-3.867	-3.920	2.750	2.673	0.003	0.003	0.010	0.010	1.000
Maternal FCGR3A+ Monocytes	0.075	0.995	-4.753	-5.196	1.801	0.000	0.001	0.000	0.005	0.000	1.000
Maternal CD14+ Monocytes	0.249	0.061	0.302	-1.271	3.399	3.538	0.018	0.009	0.018	0.012	0.198
Maternal Natural Killer Cells	0.166	0.407	2.499	2.366	1.411	1.443	0.030	0.027	0.014	0.014	0.665
Maternal B Cells	0.139	0.629	-2.642	-3.348	3.245	2.959	0.006	0.003	0.013	0.007	0.886
Maternal Plasma Cells	0.157	0.476	1.583	1.445	0.643	0.795	0.011	0.009	0.006	0.004	0.734
Maternal Naive CD8+ T Cells	0.212	0.158	1.350	2.871	3.383	1.558	0.038	0.049	0.031	0.030	0.380
Fetal Extravillous Trophoblasts	0.177	0.335	3.689	3.526	0.572	0.568	0.084	0.071	0.046	0.037	0.593

Supplementary Table 4. FGR cell type composition. The cell type composition of FGR compared to control placentas. The mean and standard deviation are reported for each group as clr-transformed and percentage cell composition. The p-values were calculated by Kolmogorov-Smirnov tests followed by Benjamini-Hochberg multiple hypothesis corrections.

Cell Type	KS Statistic	p-value	Group Mean (CLR)	Control Mean (CLR)	Group STD (CLR)	Control STD (CLR)	Group Mean (%)	Control Mean (%)	Group STD (%)	Control STD (%)	p-value Adjusted
Fetal Mesenchymal Stem Cells	0.177	0.153	3.762	3.693	0.360	0.286	0.083	0.076	0.034	0.022	0.377
Fetal CD14+ Monocytes	0.112	0.668	2.046	2.482	2.330	1.667	0.032	0.035	0.022	0.020	0.890
Fetal CD8+ Activated T Cells	0.133	0.452	3.227	3.262	1.206	0.914	0.057	0.054	0.023	0.020	0.729
Fetal Naive CD4+ T Cells	0.156	0.269	-1.669	-0.746	3.611	3.388	0.009	0.010	0.014	0.012	0.519
Fetal Naive CD8+ T Cells	0.115	0.636	-4.502	-5.196	2.094	0.000	0.001	0.000	0.004	0.000	0.886
Fetal Natural Killer T Cells	0.121	0.572	-1.343	-0.822	3.366	3.205	0.007	0.007	0.009	0.007	0.824
Fetal B Cells	0.109	0.700	3.333	3.364	0.294	0.307	0.052	0.054	0.013	0.014	0.921
Fetal GZMK+ Natural Killer	0.123	0.557	-3.005	-2.858	2.932	3.105	0.002	0.003	0.004	0.006	0.824
Fetal Memory CD4+ T Cells	0.150	0.312	3.741	3.828	0.298	0.248	0.079	0.085	0.019	0.018	0.562
Fetal Hofbauer Cells	0.212	0.052	1.811	1.500	1.947	1.816	0.020	0.015	0.016	0.011	0.186
Fetal Plasmacytoid Dendritic Cells	0.159	0.250	2.767	3.159	1.878	0.864	0.045	0.048	0.021	0.015	0.499
Fetal GZMB+ Natural Killer	0.077	0.958	-5.000	-5.130	1.076	0.690	0.000	0.000	0.001	0.001	1.000
Fetal Endothelial Cells	0.208	0.060	3.514	3.570	0.325	0.269	0.064	0.066	0.022	0.017	0.198
Fetal Syncytiotrophoblast	0.144	0.359	2.239	2.623	1.777	0.563	0.027	0.028	0.017	0.013	0.616
Fetal Fibroblasts	0.171	0.181	-1.230	-1.794	3.178	3.107	0.006	0.004	0.012	0.008	0.426
Fetal Cytotrophoblasts	0.141	0.385	5.224	5.229	0.181	0.097	0.340	0.339	0.051	0.032	0.648
Fetal Proliferative Cytotrophoblasts	0.094	0.848	-5.196	-5.196	0.000	0.000	0.000	0.000	0.000	0.000	1.000
Fetal Nucleated Red Blood Cells	0.098	0.806	-3.685	-3.827	2.105	1.868	0.001	0.000	0.002	0.000	1.000
Maternal CD8+ Activated T Cells	0.071	0.980	-1.998	-2.083	3.384	3.438	0.005	0.006	0.008	0.010	1.000
Maternal Naive CD4+ T Cells	0.114	0.652	-3.521	-3.920	3.129	2.673	0.006	0.003	0.015	0.010	0.890
Maternal FCGR3A+ Monocytes	0.094	0.848	-5.196	-5.196	0.000	0.000	0.000	0.000	0.000	0.000	1.000
Maternal CD14+ Monocytes	0.086	0.907	-0.807	-1.271	3.494	3.538	0.012	0.009	0.016	0.012	1.000
Maternal Natural Killer Cells	0.097	0.820	1.789	2.366	2.457	1.443	0.026	0.027	0.017	0.014	1.000
Maternal B Cells	0.102	0.777	-3.390	-3.348	3.160	2.959	0.005	0.003	0.011	0.007	0.999
Maternal Plasma Cells	0.221	0.038	1.167	1.445	1.078	0.795	0.008	0.009	0.005	0.004	0.140
Maternal Naive CD8+ T Cells	0.164	0.222	2.083	2.871	2.620	1.558	0.040	0.049	0.028	0.030	0.479
Fetal Extravillous Trophoblasts	0.088	0.896	3.409	3.526	1.255	0.568	0.072	0.071	0.038	0.037	1.000

Supplementary Table 5. PTD cell type composition. The cell type composition of PTD compared to control placentas. The mean and standard deviation are reported for each group as clr-transformed and percentage cell composition. The p-values were calculated by Kolmogorov-Smirnov tests followed by Benjamini-Hochberg multiple hypothesis corrections.

Cell Type	KS Statistic	p-value	Group Mean (CLR)	Control Mean (CLR)	Group STD (CLR)	Control STD (CLR)	Group Mean (%)	Control Mean (%)	Group STD (%)	Control STD (%)	p-value Adjusted
Fetal Mesenchymal Stem Cells	0.252	0.068	3.556	3.693	0.340	0.286	0.067	0.076	0.024	0.022	0.209
Fetal CD14+ Monocytes	0.370	0.001	-0.064	2.482	3.704	1.667	0.019	0.035	0.019	0.020	0.010
Fetal CD8+ Activated T Cells	0.412	0.000	3.626	3.262	0.297	0.914	0.071	0.054	0.019	0.020	0.002
Fetal Naive CD4+ T Cells	0.294	0.020	-2.296	-0.746	3.428	3.388	0.006	0.010	0.009	0.012	0.090
Fetal Naive CD8+ T Cells	0.191	0.278	-4.367	-5.196	2.270	0.000	0.002	0.000	0.011	0.000	0.527
Fetal Natural Killer T Cells	0.200	0.231	-1.655	-0.822	3.513	3.205	0.007	0.007	0.010	0.007	0.489
Fetal B Cells	0.497	0.000	2.990	3.364	0.510	0.307	0.040	0.054	0.014	0.014	0.000
Fetal GZMK+ Natural Killer	0.309	0.012	-4.009	-2.858	2.320	3.105	0.001	0.003	0.002	0.006	0.058
Fetal Memory CD4+ T Cells	0.273	0.038	3.655	3.828	0.356	0.248	0.074	0.085	0.022	0.018	0.140
Fetal Hofbauer Cells	0.412	0.000	-0.523	1.500	3.037	1.816	0.008	0.015	0.014	0.011	0.002
Fetal Plasmacytoid Dendritic Cells	0.473	0.000	3.321	3.159	1.546	0.864	0.067	0.048	0.025	0.015	0.000
Fetal GZMB+ Natural Killer	0.309	0.012	-5.196	-5.130	0.000	0.690	0.000	0.000	0.000	0.001	0.058
Fetal Endothelial Cells	0.209	0.190	3.429	3.570	0.289	0.269	0.058	0.066	0.015	0.017	0.430
Fetal Syncytiotrophoblast	0.703	0.000	-1.782	2.623	3.586	0.563	0.008	0.028	0.011	0.013	0.000
Fetal Fibroblasts	0.245	0.080	-2.382	-1.794	3.158	3.107	0.004	0.004	0.007	0.008	0.226
Fetal Cytotrophoblasts	0.524	0.000	5.063	5.229	0.266	0.097	0.294	0.339	0.061	0.032	0.000
Fetal Proliferative Cytotrophoblasts	0.309	0.012	-5.196	-5.196	0.000	0.000	0.000	0.000	0.000	0.000	0.058
Fetal Nucleated Red Blood Cells	0.455	0.000	-1.993	-3.827	2.175	1.868	0.001	0.000	0.001	0.000	0.000
Maternal CD8+ Activated T Cells	0.097	0.953	-2.167	-2.083	3.451	3.438	0.007	0.006	0.011	0.010	1.000
Maternal Naive CD4+ T Cells	0.227	0.125	-2.553	-3.920	3.368	2.673	0.006	0.003	0.017	0.010	0.322
Maternal FCGR3A+ Monocytes	0.309	0.012	-5.196	-5.196	0.000	0.000	0.000	0.000	0.000	0.000	0.058
Maternal CD14+ Monocytes	0.403	0.000	0.505	-1.271	3.414	3.538	0.022	0.009	0.022	0.012	0.003
Maternal Natural Killer Cells	0.258	0.058	1.355	2.366	2.848	1.443	0.024	0.027	0.017	0.014	0.198
Maternal B Cells	0.582	0.000	0.714	-3.348	3.205	2.959	0.020	0.003	0.017	0.007	0.000
Maternal Plasma Cells	0.330	0.006	1.171	1.445	0.560	0.795	0.007	0.009	0.004	0.004	0.034
Maternal Naive CD8+ T Cells	0.491	0.000	-1.049	2.871	4.044	1.558	0.019	0.049	0.023	0.030	0.000
Fetal Extravillous Trophoblasts	0.597	0.000	4.435	3.526	0.490	0.568	0.171	0.071	0.077	0.037	0.000

Supplementary Table 6. FGR+HDP cell type composition. The cell type composition of FGR+HDP compared to control placentas. The mean and standard deviation are reported for each group as clr-transformed and percentage cell composition. The p-values were calculated by Kolmogorov-Smirnov tests followed by Benjamini-Hochberg multiple hypothesis corrections.

Cell Type	KS Statistic	p-value	Group Mean (CLR)	Control Mean (CLR)	Group STD (CLR)	Control STD (CLR)	Group Mean (%)	Control Mean (%)	Group STD (%)	Control STD (%)	p-value Adjusted
Fetal Mesenchymal Stem Cells	0.130	0.475	3.722	3.693	0.329	0.286	0.079	0.076	0.026	0.022	0.734
Fetal CD14+ Monocytes	0.280	0.003	1.719	2.482	2.550	1.667	0.025	0.035	0.016	0.020	0.023
Fetal CD8+ Activated T Cells	0.189	0.105	3.438	3.262	0.406	0.914	0.060	0.054	0.017	0.020	0.280
Fetal Naive CD4+ T Cells	0.143	0.354	-1.321	-0.746	3.500	3.388	0.008	0.010	0.011	0.012	0.616
Fetal Naive CD8+ T Cells	0.048	1.000	-5.080	-5.196	0.901	0.000	0.000	0.000	0.002	0.000	1.000
Fetal Natural Killer T Cells	0.100	0.777	-0.797	-0.822	3.282	3.205	0.008	0.007	0.008	0.007	0.999
Fetal B Cells	0.308	0.001	3.090	3.364	1.105	0.307	0.046	0.054	0.013	0.014	0.007
Fetal GZMK+ Natural Killer	0.158	0.248	-3.567	-2.858	2.781	3.105	0.002	0.003	0.005	0.006	0.499
Fetal Memory CD4+ T Cells	0.168	0.191	3.787	3.828	0.269	0.248	0.082	0.085	0.019	0.018	0.430
Fetal Hofbauer Cells	0.277	0.004	0.497	1.500	2.588	1.816	0.011	0.015	0.011	0.011	0.025
Fetal Plasmacytoid Dendritic Cells	0.188	0.106	3.217	3.159	1.139	0.864	0.054	0.048	0.020	0.015	0.280
Fetal GZMB+ Natural Killer	0.048	1.000	-5.128	-5.130	0.528	0.690	0.000	0.000	0.000	0.001	1.000
Fetal Endothelial Cells	0.078	0.952	3.581	3.570	0.281	0.269	0.067	0.066	0.017	0.017	1.000
Fetal Syncytiotrophoblast	0.330	0.000	1.832	2.623	2.024	0.563	0.022	0.028	0.015	0.013	0.003
Fetal Fibroblasts	0.139	0.390	-2.249	-1.794	3.291	3.107	0.005	0.004	0.009	0.008	0.648
Fetal Cytotrophoblasts	0.114	0.640	5.232	5.229	0.102	0.097	0.339	0.339	0.034	0.032	0.886
Fetal Proliferative Cytotrophoblasts	0.048	1.000	-5.196	-5.196	0.000	0.000	0.000	0.000	0.000	0.000	1.000
Fetal Nucleated Red Blood Cells	0.163	0.221	-3.433	-3.827	1.974	1.868	0.000	0.000	0.000	0.000	0.479
Maternal CD8+ Activated T Cells	0.112	0.661	-2.526	-2.083	3.373	3.438	0.005	0.006	0.009	0.010	0.890
Maternal Naive CD4+ T Cells	0.095	0.829	-3.413	-3.920	3.041	2.673	0.004	0.003	0.011	0.010	1.000
Maternal FCGR3A+ Monocytes	0.048	1.000	-5.196	-5.196	0.000	0.000	0.000	0.000	0.000	0.000	1.000
Maternal CD14+ Monocytes	0.274	0.004	0.502	-1.271	2.952	3.538	0.014	0.009	0.013	0.012	0.025
Maternal Natural Killer Cells	0.092	0.854	2.282	2.366	1.617	1.443	0.027	0.027	0.014	0.014	1.000
Maternal B Cells	0.276	0.004	-1.538	-3.348	3.381	2.959	0.007	0.003	0.009	0.007	0.025
Maternal Plasma Cells	0.149	0.310	1.298	1.445	0.620	0.795	0.008	0.009	0.004	0.004	0.562
Maternal Naive CD8+ T Cells	0.231	0.025	2.221	2.871	2.363	1.558	0.038	0.049	0.027	0.030	0.103
Fetal Extravillous Trophoblasts	0.228	0.027	3.784	3.526	0.487	0.568	0.089	0.071	0.042	0.037	0.110

Supplementary Table 7. PE cell type composition. The cell type composition of PE compared to control placentas. The mean and standard deviation are reported for each group as clr-transformed and percentage cell composition. The p-values were calculated by Kolmogorov-Smirnov tests followed by Benjamini-Hochberg multiple hypothesis corrections.

Feature	Variance Inflation Factor (VIF) Female Fetuses	Variance Inflation Factor (VIF) Male Fetuses
Gestational Weeks	6.67	5.49
Pregravid BMI^2	5.53	4.03
Labor Initiation	1.65	2.06
Smoker	2.19	2.03
Illicit Drug User	2.21	2.06

Supplementary Table 8. Variance inflation factors for GLM features in female and male fetuses.

	Control n=30	FGR n=30	FGR+HDP n=30	PE n=30	PTD n=30
**Edges (n)**	1	10	80,612	4,032	3,020
**Nodes (n)**	2	17	714	1625	1161

Supplementary Table 9. Network and partition quality metrics and overview after downsampling. Random downsampling was performed to select thirty placentas for each condition. Then interomics correlations followed by Bonferroni correction as described. The number of edges and nodes participating in significant interomics correlations are reported by condition.

Library or Language	Version	Citation	Documentation
Jupyter	6.5.4	Kluyver et al. 2016[2]	https://jupyter.org/
logging	0.5.1.2	open source	https://docs.python.org/3/library/logging.html
matplotlib	3.8.2	Hunter et al. 2007[3]	https://matplotlib.org/
networkx	3.1	Hagberg et al. 2008[4]	https://networkx.org/
nxviz	0.7.3	open source	https://pypi.org/project/nxviz/
numpy	1.24.3	Harris et al. 2020[5]	https://numpy.org/
pandas	2.0.1	Reback et al. 2020[6]	https://pandas.pydata.org/
python	3.11.6	open source	https://docs.python.org/release/3.11.6/
pyvis	0.3.1	open source	https://pyvis.readthedocs.io/en/latest/documentation.html
scipy	1.10.1	Virtanen et al. 2020[7]	https://scipy.org/
seaborn	0.12.2	Waskom et al. 2021[8]	https://seaborn.pydata.org/index.html
sklearn	1.2.2	Pedregosa et al. 2011[9]	https://scikit-learn.org/stable/index.html
statsmodels	0.14.0	Seabold & Perktold 2010[10]	https://www.statsmodels.org/stable/index.html

Supplementary Table 10. Key resources: software and algorithms.

Supplementary References

1. Campbell K.A., et al. Placental cell type deconvolution reveals that cell proportions drive preeclampsia gene expression differences. *Commun Biol*. **6**(1), 264 (2023).

2. Kluyver T, et al. Jupyter Notebooks – a publishing format for reproducible computational workflows. *In: Loizides F, Scmidt B, editors. Positioning and Power in Academic Publishing: Players, Agents and Agendas*. 87-90; 10.3233/978-1-61499-649-1-87 (2016).

3. Hunter J.D. Matplotlib: A 2D graphics environment. *Comput Sci Eng*. **9**(3), 90-95 (2007).

4. Hagberg A.A., Schult D.A., & Swart P.J. Exploring network structure, dynamics, and function using NetworkX. *In: Varoquaux G, Vaught T, Millman J, editors. Proceedings of the 7th Python in Science Conference (SciPy 2008).* 11-15 (2008).

5. Harris C.R., et al. Array programming with NumPy. *Nature*. **585**(7825), 357-362 (2020).

6. Reback J., et al. pandas-dev/pandas: Pandas 2.0.1. *Zenodo*. 10.5281/zenodo.3715232 (2020).

7. Virtanen P., et al. SciPy 1.0: Fundamental algorithms for scientific computing in Python. *Nat Methods*. **17**(3), 261-272 (2020).

8. Waskom M.L. seaborn: statistical data visualization*. J Open Source Softw*. **6**(60), 3021 (2021).

9. Pedregosa F., et al. Scikit-learn: Machine learning in Python*. J Mach Learn Res*. **12**, 2825-2830 (2021).

10. Seabold S, Perktold J. Statsmodels: Econometric and statistical modeling with Python. *In: Proceedings of the 9th Python in Science Conference (SciPy 2010)*. 92-96, 10.25080/Majora-92bf1922-011 (2021).
