# Supplementary material for: Placental network differences among obstetric syndromes identified with an integrated multiomics approach
Source: Commun Biol. 2025 Aug 18;8:1239. doi: 10.1038/s42003-025-08631-6 (PMC12361440; doi:10.1038/s42003-025-08631-6)
Supplement: Supplementary file 9 — Description of Additional Supplementary Files [file 42003_2025_8631_MOESM9_ESM.docx]

Description of Additional Supplementary Files

**File name:** Supplementary Data 1

**Description:** GLMs for female fetuses. For each analyte the following model was fit: analyte ~ GestationalWeeks + PregravidBMI2 + C(LaborInitiation) + C(Smoker) + C(IllicitDrugUser). Anderson-Darling tests were used to evaluate the distribution of the data. Z-score was used to detect outliers with the threshold set as the absolute value of three, if more than 2.5% of all measurements surpassed this threshold then the analyte was declared an outlier. If the analyte had a non-normal distribution and/or the analyte was an outlier than a Gamma family was used to train the GLMs, otherwise a Gaussian family was Page 8 of 24 applied. Benjamini-Hochberg multiple hypothesis adjustment was performed. The β-coefficients for the variables, y-intercepts, p-value, adjusted pvalue, and pseudo R-squared value for each GLM were reported.

**File name:** Supplementary Data 2

**Description:** GLMs for male fetuses. For each analyte the following model was fit: analyte ~ GestationalWeeks + PregravidBMI2 + C(LaborInitiation) + C(Smoker) + C(IllicitDrugUser). Anderson-Darling tests were used to evaluate the distribution of the data. Z-score was used to detect outliers with the threshold set as the absolute value of three, if more than 2.5% of all measurements surpassed this threshold then the analyte was declared an outlier. If the analyte had a non-normal distribution and/or the analyte was an outlier than a Gamma family was used to train the GLMs, otherwise a Gaussian family was applied. Benjamini-Hochberg multiple hypothesis adjustment was performed. The β-coefficients for the variables, y-intercepts, p-value, adjusted pvalue, and pseudo R-squared value for each GLM were reported.

**File name:** Supplementary Data 3

**Description:** Control interomics communities. For each node of the control Page 9 of 24 interomics network, we report the community to which it belongs along with its closeness centrality score, which was normalized to a scale of 0 to 1 for each community.

**File name:** Supplementary Data 4

**Description:** FGR interomics communities. For each node of the FGR interomics network, we report the community to which it belongs along with its closeness centrality score, which was normalized to a scale of 0 to 1 for each community

**File name:** Supplementary Data 5

**Description:** PTD interomics communities. For each node of the PTD interomics network, we report the community to which it belongs along with its closeness centrality score, which was normalized to a scale of 0 to 1 for each community.

**File name:** Supplementary Data 6

**Description:** FGR+HDP interomics communities. For each node of the FGR+HDP interomics network, we report the community to which it belongs along with its closeness centrality score, which was normalized to a scale of 0 to 1 for each community.

**File name:** Supplementary Data 7

**Description:** PE interomics communities. For each node of the PE interomics network, we report the community to which it belongs along with its closeness centrality score, which was normalized to a scale of 0 to 1 for each community.
